# Predictors of response to family-based treatment for anorexia nervosa in youth: insights from the VIBUS project

**DOI:** 10.1007/s00787-025-02766-x

**Published:** 2025-06-11

**Authors:** Mette Bentz, Signe Holm Pedersen, Ulla Moslet, Nikolaj Petersen, Anne Katrine Pagsberg

**Affiliations:** 1 Child and Adolescent Mental Health Center, Mental Health Services in the Capital Region of Denmark, Bispebjerg Bakke 30, 2400 Copenhagen, NV Denmark; 2 Omicron ApS, Telefonvej 8 d, 2860 Søborg, Denmark; 3 Department of Clinical Medicine, Faculty of Health and Medical Sciences, University of Copenhagen, Copenhagen, Denmark

**Keywords:** Anorexia Nervosa, Family-Based Treatment, Predictors of outcome, Children and adolescents, Treatment response, Early risk factors

## Abstract

Anorexia nervosa (AN) is a severe psychiatric disorder often emerging during adolescence, with physical, psychological, and social consequences. Family-based treatment (FBT) is the first-line intervention for young persons (YP) with AN, empowering parents to manage their child’s renourishment. Despite its benefits, less than half of YPs achieve full recovery by the end of treatment. Previous predictors, such as early weight gain, cognitive rigidity, and family dynamics, have been linked to poor outcomes, but further research is needed to refine treatment strategies. This study aimed to evaluate predictors of treatment outcomes in a large cohort consisting of 653 YP undergoing FBT for AN. Three key outcomes were examined: weight gain trajectories, time to successful treatment completion, and the risk of intensified treatment (inpatient or day patient care). The study was conducted as part of the VIBUS project, a prospective, naturalistic case series. Weight gain occurred primarily in the first three months of treatment. Older age and comorbid behavioural or emotional disorders predicted less weight gain. YPs with lower baseline BMI, higher eating disorder severity, family adversities, or comorbid conditions took longer to complete treatment, with BMI at four weeks emerging as a key predictor. Lower baseline BMI and reduced maternal renourishment capacity increased the risk of intensified treatment. These findings highlight early risk factors for suboptimal FBT response, suggesting the need for targeted interventions to improve outcomes and reduce reliance on more intensive treatments. Clinical trial registration: ClinicalTrials.gov Identifier: NCT05956366, register date 2023–07-13.

**Supplementary Information:**

The online version contains supplementary material available at 10.1007/s00787-025-02766-x.

## Background

Anorexia nervosa (AN) is a serious psychiatric disorder, often emerging in adolescence, with severe impacts on both the young person´s (YP) physical and psychological development, and significant caregiver burden and societal cost [49]*.* The globally recommended first-line treatment for YPs with AN is outpatient family-based treatment (FBT), where parents are supported in taking responsibility for YP’s renourishment [17, 21, 31]. While FBT has improved recovery rates, less than half of YP achieve full recovery by the end of treatment (EOT) [29, 41]. Factors such as limited weight gain by four weeks of FBT, cognitive inflexibility, and criticism between family members have been linked to poorer outcomes [14], but it remains unclear whether other markers or combination of markers could better predict those in need of treatment modifications.

To improve future success rates, it is vital to understand the characteristics and treatment needs of YPs for whom FBT is insufficient. The timing of improvement and early identification of non-responders is crucial, as evidence indicates that longer illness duration reduces recovery chances [48]. Furthermore, reducing hospitalization is important, as intensified treatment may be associated with risk of relapse [39].

In a large consecutive cohort this study examined predictors of three key treatment outcomes: Weight gain, treatment duration and the risk of need for treatment intensification, including hospitalization. Due to the exploratory nature of this study with no control condition, potential predictors may be non-specific treatment factors that could influence course of treatment for YPs with AN regardless of which treatment they were offered. As family-based approaches are recommended internationally for YPs with AN, it will nonetheless increase our understanding of what characterizes YPs with less optimal outcome.

## Aims

First, we aimed to assess whether YP or family characteristics at start of treatment influence weight gain trajectories during FBT for AN during the first year (analysis 1).

Second, we aimed to assess whether YP or family characteristics at start and after 4 weeks of treatment may predict time to successful completion of treatment for AN in YPs within an observation period of 2 years (analysis 2a & 2b).

Third, we aimed to characterize predictors for inpatient or day patient treatment during the course of AN treatment for YPs (analysis 3).

## Methods

The VIBUS project is an investigator-initiated, uncontrolled, prospective naturalistic case series. Clinical data is registered in structured form by clinicians. If research consent is obtained, the clinical data is entered into a research database in RedCAP [16], hosted by the capital Region of Denmark.

### Intervention

FBT [31] is offered to all YPs under the age of 18 years who live with a parent or other caregiver. Staff were initially trained by FBT manual author J. Lock [31] and have since received further training and supervision from the Maudsley Clinic in London, UK. The current treatment model of the Maudsley Clinic (FT-AN) has evolved since the initial FBT conceptualization [6]; it describes four instead of three phases, the first phase focusing on building alliance and case formulation. Among other differences are conceptualizing parent-led renourishment as love and care rather than control, and room for individualizing themes and duration in later stages to meet the needs of each family. However, both forms of family treatment for AN share the guiding principles, including parent empowerment and prioritizing renourishment and behaviour change [15]. The treatment unit where the present cohort is recruited adheres to the three original phases, and to a strong focus on initial weight gain and parent empowerment. A few local adaptations deserve mention: participants undergo FBT for AN with their parents free of charge with no fixed duration and no fixed number of sessions. This differs from the fixed 10–20 session format of manualized FBT and acknowledges the finding that a substantial subgroup is not remitted within this time frame and needs continued parent-led intervention, and previous findings suggest that continued FBT may have beneficial effects after 20 sessions [50]. In order to secure progress despite the open end of FBT, the unit has fixed time points of status and clinical rounds where clinician and family review status, which is not part of the FBT manual. The status procedure also has the function of internal treatment fidelity measure with monitoring of progress and team strategizing for dealing with family and therapy factors hindering progress. Second, YPs may be referred to eating-disorder specialized psychiatric units for day- and inpatient treatment in cases of non-response, deterioration, or acute somatic risk. This is determined on a case-by-case basis. Specifics of local implementation is described elsewhere [1].

### Participants

**Fig. 1:**
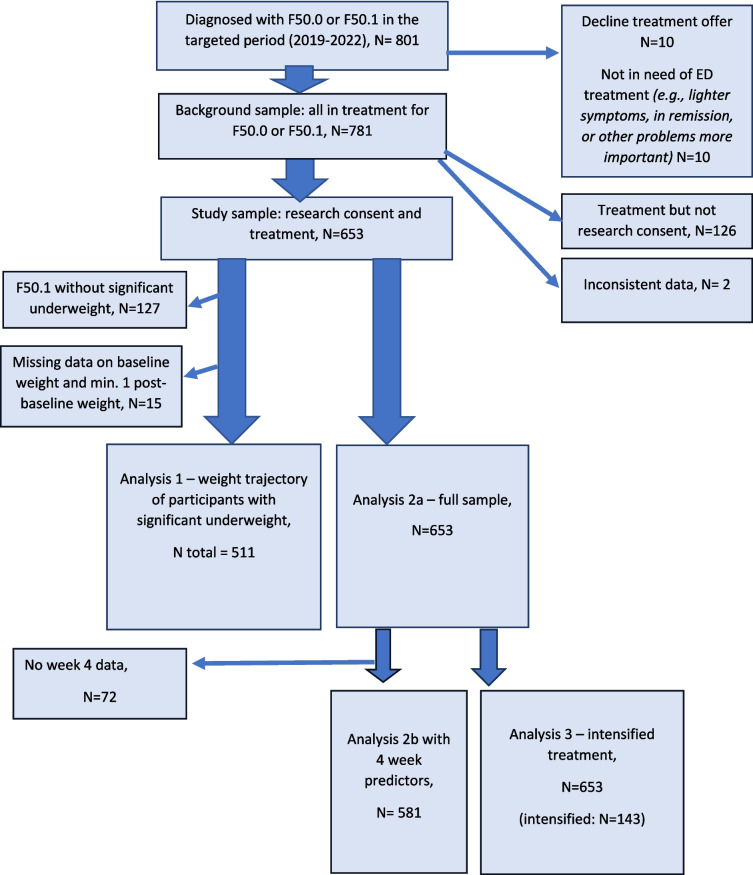
Flowchart of inclusion of participants in analyses

This study is part of the research project “*Effectiveness of family-based intervention in a child and adolescent mental health service for children and adolescents with eating disorders*” (VIBUS) which aims to identify needs and subsequently test potential improvements of standard treatment for young persons with eating disorders. VIBUS invites all YPs under the age of 18 years presenting for treatment for an eating disorder at the Child and Adolescent Mental Health Center in the Capital Region of Denmark. Participation requires written, informed consent, and the overall recruitment rate is > 80% (Fig. 1).

We included patients with typical or atypical AN (ICD-10 diagnosis of F50.0 or F50.1 [52]), in their first course of treatment, between January 2019 through December 2022. In addition to ICD-10 classification, which was in use at the time of inclusion in the study, Table 1 shows the distribution of diagnoses according to ICD-11 [53]. Participants were followed until end of treatment or April 2024, allowing a minimum of 16 months of treatment time for the latest included participant.

**Table 1:** Baseline descriptives for study sample and basic comparisons with background sample on few key variables

	Study sample, *N* = 653	Background sample, *N* = 128	difference (*p*)
Age: Mean, SD (range)	*14.4, 1.7 (7.75–17.75)*	*15.1, 1.9 (8.5–17.75)*	***t (779)* = *2.365, p* < *0.001***
Sex: m/f	*7%/93%*	*10%/90%*	*Pearson Chi-square: p* = *0.15*
Diagnose ICD-10: F50.0: N, %	*365, 55.9%*	*68, 53.1%*	*Pearson Chi-square: p* = *0.56*
Diagnose ICD-10: F50.1: N, %	*288, 44.1%*	*60, 46.9%*	*Pearson Chi-square: p* = *0.56*
Atypical AN (F50.1) due to smaller weight loss: *N*, % of F50.1	*127, 19.4%*		
Atypical AN (F50.1) due to less avoidance of fattening foods: *N*, % of F50.1	*8, 1.2%*		
Atypical AN (F50.1) due to no marked dread of being fat: *N*, % of F50.1	*74, 11.3%*		
Atypical AN (F50.1) due to no endocrine disturbances (i.e., girls still menstruating): *N*, % of F50.1	*117, 17.9%*		
Atypical AN (F50.1) due to presence of bulimic symptoms: *N*, % of F50.1	*41, 6.3%*		
Binging and/or purging behaviors: both/only binging/only purging	*4%/8%/12%*		
ICD-11: 6B80.0 Anorexia Nervosa: *N*, %	*482, 73.8%*		
ICD-11: 6B8Y other specified feeding and eating disorders (OSFED): N, %	*171, 36.2%*		
ICD-11: 6B80. × 0 Restricting pattern	*465, 71.2%*		
ICD-11: 6B80. × 1 Binge-Purge pattern	*188, 28.8%*		
Relative BMI, mean, SD (range)	*0.85, 0.11 (0.58–1.33)*		
EDE global score, mean, SD (range)	*3.04, 1.4 (0–6)*		
Any comorbid diagnoses, *N*, %	*187, 28,6%*		
More than one comorbid diagnosis, *N*, %	*74, 11.3%*		
Autism spectrum (ICD-10: F80-88): *N*, %	*136, 20.8%*		
Anxiety disorder (ICD-10: F40-48): *N*, %	*53, 8.1%*		
Behavioral emotional disorder (ICD-10: F90-98): *N*, %	*49, 7.5%*		
Affective disorder (ICD-10: F30-38): *N*, %	*34, 5.2%*		
Other comorbidity or intellectual disability, *N*, %	*20, 3.1%*		
Intensified treatment at any time point (day patient/inpatient/both): *N*, %	*143 (21,9%)*		

The background sample constitutes YPs starting treatment for F50.0 or F50.1 in the study period who were not included in the study sample due to lack of research consent or inconsistent data. Conversion to ICD-11 diagnoses is approximated: a weight loss of 15% below expected would for most YP entail a weight under 5 th percentile and thus constitutes significantly low weight according to ICD-11, and we therefore classified all with ICD-10 diagnosis of 50.0 and those with ICD-10 diagnosis of F50.1 due no endocrine disturbances (i.e.; girls still menstruating) as 6B80.0 Anorexia Nervosa according to ICD-11. Comorbid diagnoses of intellectual disability (F70-78 or R41.8), autism spectrum conditions (F80-F88) and behavioral emotional disorders (F90-F98) were included regardless of when it was assigned, as they represent long-lasting developmental conditions. All other diagnoses were considered only if they were assigned before intake or by 4 weeks.

Legend: *N* Number; *SD* Standard deviation; *p* Significance level, ICD-10 = WHO´s International classification of Diseases, 10 th edition, F50.0 = anorexia nervosa, F50.1 = atypical anorexia nervosa, ICD-11 = WHO´s International classification of Diseases, 11 th edition, BMI = Body mass index, relative BMI = actual BMI divided by the population-based median BMI for sex and age, EDE = Eating Disorder Examination, global EDE = global score of psychological symptoms derived from the EDE

### Measures

#### Potential predictors

All participants undergo an Eating Disorder Examination (EDE) [12], assessing eating disorder symptoms. They further undergo a somatic evaluation, and parents are interviewed on YP development, symptoms, and family life. We included the intake diagnosis and basis for an atypical diagnosis, the degree of underweight (relative BMI for age and sex), the global score from the EDE (EDE-global), patient-reported duration of restrictive eating, and presence of compulsive exercise, binging or purging as AN-related predictors. As non-AN-specific predictors we included age, sex, comorbidity and caregiver status (living together or apart, one or 2 parents involved in daily care). Moreover, parental reports of a range of adversities for the YP prior to the onset of AN, and of practical, economic, or health-related adversities in the family, and somatic and mental illness in parents were registered in a structured, binominal form (present/not present) during the parent interview at intake. Among adversities registered for the YP was somatic or mental health challenges, a history of overweight, losses, bullying or other peer relation adversities. Among adversities in the family were major transitions or trauma affecting the family, health challenges in a sibling or a parent, and practical or economic challenges (online resource 1). Lastly, clinician´s and parents´ own assessment of parents´ ability to take an active role in renourishment were included; parents were asked directly, and their answers registered by the clinician.

Comorbid diagnoses were registered if assigned by standard clinical assessment either prior to or during AN treatment. Clinical assessment includes K-SADS-PL interviews [3], and if indicated Autism Diagnostic Interview-Revised [34] or other relevant assessments of psychopathology. Diagnoses F80-F88 (autism spectrum conditions) and F90-F98 (behavioural or emotional disorders) was included regardless of when it was assigned, as they represent long-lasting developmental conditions. All other diagnoses were considered only if they were assigned before intake or by 4 weeks after intake.

The outpatient unit has a routine of “taking stock” and evaluate progress with each family at 4 weeks from start (5 th therapy session), 3 months from start and every 3 months until the final stage of treatment, and at treatment termination. A structured status questionnaire is filled by family and therapist in collaboration (Online Resource 1). It´s content includes single items from the EDE as well as clinician´s assessment of moderating and mediating factors for treatment progress. Information from 4-week status (analysis 2b) included as potential predictors was: Relative BMI, information on menstruation, binging, purging and excessive exercising behaviours, clinician´s assessment of patient motivation for change and of collaborative relation between patient, family and treatment team assessed on a 4-point Likert-scale, clinician assessment of illness-maintaining and supporting therapy factors, diagnostic questions from EDE, and information on eating responsibility, school and furlough (Online Resource Table 2b). Each of these review points are followed by a clinical round with the broader treatment team to ensure alignment and model fidelity.

#### Outcome measures

Outcome of analysis 1 (weight trajectories) was change in relative BMI. Relative BMI is calculated as actual BMI divided by expected BMI. Expected BMI is the median BMI for sex and age according to Danish population norms [47].

Outcome of analyses 2a & 2b was time to successful treatment completion. This is defined as a collaborate assessment between treatment team and family and reflects the appraisal that the young person is well, and that the family can manage potential residual symptoms without further treatment*.* In the present study we viewed treatment completion in a strict sense not counting cases who ended eating disorder treatment but needed referral for assessment and/or treatment of other psychiatric challenges. Therefore, forms of termination were successful completion, drop out, and transfer to other kinds of treatments for either eating disorder or for comorbidity.

Outcome of analysis 3 was one or more admissions to day program or inpatient unit (intensified treatment) at any time during the course of treatment for AN.

#### Supplementary measures of remission

We report three descriptive indicators of remission, two behavioural indicators: a) age-appropriate responsibility for eating, and b) mild to no intention of dietary restraint; and one psychological indicator (Table 2).

**Table 2:** Indicators of remission by end of treatment for participants with successful treatment completion and participants with other ways of ending treatment or not ended in observation period

	Participants with successful treatment completion within the observation period, *N* = 351 (53.8%)						Participants not completing treatment successfully within the observation period, *N*** = **302 (46.2%)					
Indicators of remission	Mean	SD	q25	median	q75	N missing data	Mean	SD	q25	median	q75	N missing data
Relative BMI for age and sex by end of treatment	0.99	0.09	0.94	0.98	1.04	17	0.88	0.28	0.87	0.94	1.01	26
Difference between start and end of treatment in relative BMI for age and sex	0.13	0.08	0.07	0.13	0.18	17	0.03	0.29	0.04	0.10	0.16	26
Age-appropriate responsibility for eating*	3.79	0.58	4.00	4.00	4.00	105	3.22	1.06	3.00	4.00	4.00	184
Level of psychological symptoms**	1.39	1.11	0.67	1.33	2.00	113	3.06	1.58	2.00	3.00	4.33	196
If ended treatment in any forms: Duration (months) in CAMHS	10.21	5.91	6.05	9.57	13.09	0	10.49	7.67	4.46	8.78	14.75	0
If ended treatment in any forms: Number of sessions in outpatient CAMHS	19.11	10.13	12.00	17.00	25.00	22	19.90	13.22	9.00	17.00	28.00	71

Other ways of ending treatment besides successful completion: dropout, referral to eating disorder support in primary sector, referral to other units of Child and Adolescent Mental Health Centre for comorbidity, or referral to adult mental health services for treatment of eating disorder or comorbidity

Legend: *SD* Standard deviation, q25 = 25 th quartile, q75 = 75 th quartile, BMI = body mass index, relative BMI = actual BMI divided by the population-based median BMI for sex and age, CAMHS = Child and Adolescent Mental Health Service. *Age-appropriate responsibility for eating is evaluated on a 4-point Likert scale (Parents have full responsibility for eating/Patient has growing responsibility for eating in limited areas/Patient has co-responsibility or is practicing increasing responsibility with support from parents/patient has main responsibility for eating (corresponding to the normal for his/her age)

**Level of psychological symptoms: mean of three EDE items scored on a 7-point Likert scale: Importance of shape, Importance of weight, and Feeling fat

We include a) age-appropriate responsibility for eating because FBT initially and temporarily places responsibility on parents, and the return of responsibility to the YP is an important later treatment focus and indicator for remission. Age-appropriate responsibility for sufficient and regular eating is reported collaboratively by the therapist and the family via a 4-point Likert scale (*Parents have full responsibility for eating/Patient have growing responsibility for eating in limited areas/Patient have co-responsibility or is practicing increasing responsibility with support from parents/patient have main responsibility for eating (corresponding to the normal for his/her age)).*

Intention of dietary restraint is estimated from the following questions with wordings from the EDE-child interview: Restraint over eating, frequency (*Over the past four weeks, have you deliberately been trying to cut down on what you eat?*), Desire to lose weight, frequency (*over the past four weeks, have you wanted to lose weight?*), and Maintained low weight *((if low weight) Have you been trying to make sure that you do not put on any weight?(yes/no))*. We defined mild to no dietary restraint as no intention of weight loss, no intention to maintain underweight, and experienced intention of restrictive eating less than 50% of days for the last four weeks.

As a measure of psychological symptoms we report the mean of three EDE items scored on a 7-point Likert scale (where 0 represent the lowest and 6 the highest intensity or frequency as per EDE instructions [12]): Importance of shape, Importance of weight, and Feeling fat. This is a crude estimation of level of psychological AN symptoms in lieu of a full EDE-interview at treatment termination.

### Statistical procedures

A statistician independent of the data collection site (NP) carried out the statistical analyses. As this is primarily a descriptive and hypothesis generating study with no control condition potential predictors may be non-specific treatment factors. This is reflected in the statistical methodology, as no adjustment for multiplicity has been made, allowing for sequential testing of potential predictors at a significance level of α =0. 0.05 supporting the exploratory nature of this study.

#### Analysis 1: weight trajectories

The analysis included weight observations at baseline, week 4 and months 3, 6, 9 and 12 of treatment. Participants diagnosed with AAN, who were not significantly underweight (i.e., not meeting the F50.0 criterion of ≥ 15% weight deficit) were excluded.

Latent class analysis was applied to relative BMI changes in a mixed model with a participant-specific random effect over time, aiming to identify the number of latent classes of trajectories. Based on Akaike Information Criteria (AIC) and cross-validation, a single latent class best fit the data.

Subsequently, a mixed model for repeated measures (MMRM) with a participant specific random effect and a fixed effect from each visit as a base model. Covariates were initially screened using an $\alpha =0.1$ threshold with non-significant variables excluded. Significant variables had missing values imputed, either by random sampling or assigning a “neutral” value based on clinical input. All significant variables were added to the base model, and iterative ANCOVA F-test removed the least significant variable, until all covariates achieved $\alpha =0.05$ significance. Pairwise interactions among the remaining covariates and between each covariate and the time points were tested.

#### Analysis 2a: time to successful treatment completion

To analyze the time from treatment-start to successful treatment completion we employed survival analysis, with other forms of treatment termination considered censored. Censoring allows the inclusion of incomplete data, accounting for potential treatment completion outside the observation window (e.g., after transfer from FBT).

We conducted a model selection process, with a Cox proportional hazards model, and a missing value imputation process, both similar to the process from analysis 1.

Remaining covariates were included in a single model, iteratively refined by ANCOVA Chi-squared tests. Pairwise interaction effects among the remaining covariates were tested.

#### Analysis 2b: week 4 variables as additional covariates

We repeated analysis 2a, measuring time from the 4-week visit to the event. Only participants with data on 4-week weight and a minimum of one later weight was included (Fig. 1). The model was re-fitted using the new population. Before proceeding, we tested if both 4-week relative BMI and baseline relative BMI were significant for successful treatment completion (alpha = 0.05) within the same model.

Four-week variables was individually added to the model, excluding non-significant variables ($\alpha =0.05$). After imputing missing values as before, remaining covariates were combined with those from baseline time-to-event analysis. Iterative exclusion of non-significant covariates (based on ANCOVA Chi-squared tests yielded a final model.

#### Analysis 3: predictors of intensified treatment

Logistic regression was employed to test predictors of admission to day program or inpatient unit. Only predictors observed at baseline were tested.

The model selection process followed the previously described process: Initial individual testing, missing value imputation, iterative ANCOVA testing with backwards **elimination** and interaction testing.

## Results

Of 801 YPs diagnosed in the time period, 781 (97.5%) started AN treatment, where FBT is offered as the standard treatment. Of these, 653 (83.6%) gave informed research consent and consistent data and therefore constituted the study sample for the present study. The study sample was slightly but significantly younger than those starting treatment without research consent (the background sample), with a mean difference of 0.67 years (95% confidence interval 0.35–0.99 years). Distributions of sex and diagnosis (AN/AAN) were similar between study sample and background sample (Table 1).

*N* = 351 (53.8%) achieved successful treatment completion during the study period (Tables 2 and 3). *N* = 64 (9.8%) dropped out of treatment, *N* = 106 (16.2%) were transferred to other treatment for eating disorder (primary sector services, moved and continued in another region, or Adult Mental Health Service), *N* = 116 (17.8%) were transferred for assessment and/or treatment of comorbid disorder (not eating disorder), and *N* = 16 (2.5%) had not ended treatment within the study period. Those who completed treatment successfully, had a higher mean relative BMI (approx. median for age and sex), and a lower level of psychological symptoms by end of treatment compared with participants with other modes of ending treatment. It is a goal for successful FBT that the patient fully regain age-appropriate eating responsibility, but their mean level of responsibility was slightly below the score representing full eating responsibility and was only slightly better than the remaining participants, indicating that some continued to need a degree of parental support even though weight and AN-cognitions were normalized.

**Table 3:** Descriptions of intensification during treatment and dietary restraint by end of treatment

Treatment description	Participants with successful treatment completion within the observation period, *N* = 351 (53.8%)			Participants not completing treatment successfully within the observation period, *N* = 302 (46.2%)		
	*N*	ratio	*N* missing data	*N*	ratio	*N* missing data
No intensified treatment	317	0.90	0	193	0.64	0
First intensification of treatment = day program	13	0.04	0	55	0.18	0
First intensification of treatment = Inpatient treatment	21	0.06	0	54	0.18	0
No dietary restraint by EOT*	189	0.54	29	43	0.14	24
Dietary restraint present by EOT*	155	0.44	29	241	0.80	24

Legend: *If ended treatment in any forms: Mild to no dietary restraint defined as: as no intention of weight loss, no intention to maintain underweight, and experienced intention of restrictive eating less than 50% of days for the last four weeks. *EOT* End of treatment

### Analysis 1: predictors of weight gain trajectories

Analysis 1 aimed to assess whether YP or family characteristics at intake influenced weight gain trajectory. Following the predefined criteria of significant underweight, the analysis includes a cohort of 511 participants (Fig. 1). Frequencies, significance level, and effect sizes of all the individually tested variables can be found in Online Resource 2.

There was a clear increase in relative BMI throughout the treatment in the overall model taking the remaining significant covariates into account. Expected marginal means of each time point showed that most of the weight increase were achieved within the first 4 weeks (adds 0.05 to the relative baseline BMI) and 3 months (adds 0.12 to the relative baseline BMI), while data from 6- and 9-months follow-up only added little weight gain (Table 4).

**Table 4:** Expected marginal means of relative BMI at each time point

Time	em mean	SE	lower CL	upper CL
4 weeks	0,05	0,00	0,05	0,06
3 months	0,12	0,01	0,11	0,13
6 months	0,13	0,01	0,12	0,14
9 Months	0,14	0,01	0,12	0,15
12 Months	0,13	0,01	0,12	0,15

Expected marginal means (em mean) represent the model-based increase from baseline in relative BMI when taking the remaining significant covariates into account

Legend: *EM Mean* expected marginal means; *SE* Standard error; *CL* Confidence interval limit

Further, there was an interaction between time points and baseline relative BMI showing that the higher rates of increase in relative BMI earlier in the treatment phase were enhanced with lower levels of baseline relative BMI.

**Fig. 2:**
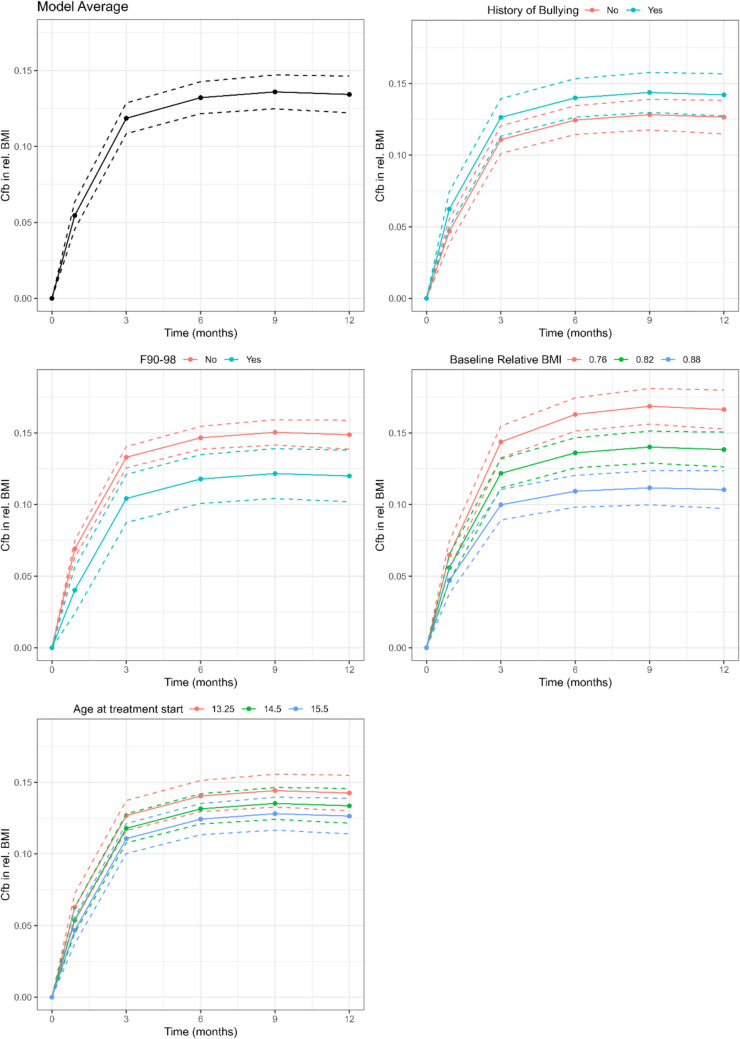
Analysis of weight trajectory (analysis 1). Note: Models show the expected marginal means with 95% confidence in each group at each time point. Expected marginal means are the model-based averages of weight gain, adjusted for other factors in the model. For the two numerical covariates (age and baseline relative BMI**)**, expected marginal means are shown at the covariate levels 25- quartile, median and 75- quartile

The final model estimated that when all other variables were considered, YPs with higher age at start, or higher baseline relative BMI, or a comorbid diagnosis of behavioural or emotional disorder (ICD-10: F90-98) had a smaller increase in relative BMI through the first year (negative estimates) (Table 5 and Fig. 2). Moreover, participants had a larger increase (positive estimates) in relative BMI if parents reported that the child had experienced bullying prior to debut of AN. Also of interest, all variables related to the family lost significance in the final model, implying that they did not significantly predict weight gain trajectory when the remaining baseline variables were considered.

**Table 5:** Mixed model for repeated measures analysis of weight trajectory (analysis 1), covariates in the final model in addition to effect of the time points

Covariates	Reference group	Estimate group	Estimated effect	standard error of estimate	lower CI limit	upper CI limit
Relative BMI start of treatment	0.8	0.9	−0.04	0.00	−0.04	−0.03
A history of bullying prior to debut of AN	Yes	No	−0.02	0.01	−0.03	0.00
Behavioral or emotional disorder (ICD-10: F90-98)	Yes	No	0.03	0.01	0.01	0.05
Age at start	14	15	−0.01	0.00	−0.01	0.00

The estimated effect is the change in effect in the estimate group compared to the reference group. A negative estimated effect means that the observed change in BMI was smaller in the estimate group than in the reference group

Legend: *CI* Confidence interval; *BMI* Body mass index; relative BMI = actual BMI divided by the population-based median BMI for sex and age, *AN* Anorexia nervosa, ICD-10 = WHO´s International Classification of Diseases, 10 th edition

### Analysis 2a: baseline predictors of time to successful treatment completion

Analysis 2 aimed to assess whether YP or family characteristics at intake predict time to successful treatment completion, and thus which factors are associated with the probability of successful treatment termination at any given time, and what proportion of patients reach successful treatment termination by 12 months given the influential covariates. The full study sample was included. The final model estimated that the median treatment time was 12.6 months with 51% chance of still being in treatment after 12 months. Significance and effect sizes of all the individually tested variables can be found in Online Resource 3.

#### AN-specific factors

The final model estimated that a diagnosis of atypical AN predicted shorter time to successful treatment completion; median completion time was 11.3 months for those with atypical AN compared to 13.5 months for those with typical AN (Table 6).

**Table 6:** Hazard Ratios (HR), median completion time and 1 year probability for still being in treatment (Analysis 2a)

Covariates	Level	N in level	N in level with succesful completion	*p*-value	HR estimate	lower confidence interval	upper confidence interval	median completion time	probability of still being in treatment by 12 months
Model average	all	653	351					12.57	0,51
Type of AN	F50.0	365	189		Reference			13.49	0,55
Type of AN	F50.1	288	162	0.015	1.33	1.06	1.68	11.28	0,46
Practical, economic, or health-related adversities in the family	No	614	335		Reference			12.20	0,50
Practical, economic, or health-related adversities in the family	Yes	39	16	0.022	0.55	0.33	0.92	18.03	0,67
Mental health issues in child prior to AN debut	No	575	328		Reference			11.88	0,50
Mental health issues in child prior to AN debut	Yes	78	23	0.004	0.18	0.06	0.58	17.83	0,66
Autism spectrum (ICD-10: F80-88)	No	517	305		Reference			11.55	0,48
Autism spectrum (ICD-10: F80-88)	Yes	136	46	< 0.001	0.54	0.39	0.75	17.57	0,66
Behavioral or emotional disorder (ICD-10: F90-98)	No	604	339		Reference			11.78	0,49
Behavioral or emotional disorder (ICD-10: F90-98)	Yes	49	12	0.001	0.37	0.20	0.66	24.67	0,76
EDE global score		653		< 0.001	0.85	0.78	0.92		
relative BMI, start of treatment		653		< 0.001	10.35	3.61	29.70		
Interaction effect between EDE global score and mental health issues in child prior to AN debut, if no mental health issues		575	328		Reference				
Interaction effect between EDE global score and mental health issues in child prior to AN debut if Yes to mental health issues		78	23	0.027	1.44	1.04	2.00		

Legend: *HR* Hazard ratio; *AN* Anorexia nervos; ICD-10 = WHO´s International Classification of Diseases, 10 th edition; *EDE* Eating Disorder Examination; *EDE* Global score = global score of psychological symptoms derived from the EDE, *BMI* Body mass index, relative BMI = actual BMI divided by the population-based median BMI for sex and age

The hazard ratio of EDE-global was 0.85, indicating a decrease in risk of 15% per unit increase in EDE-global. The notion of risk in this context is describing the chance of successful treatment completion, and it is quantified by the hazard function from a cox proportional hazards model. In other words, an increase in EDE-global of one unit was associated with a 15% decrease in chance of completing the treatment successfully over the course of the study timeframe. However, there was an interaction between EDE and mental health issues in child prior to AN debut, so that for participants with these issues, the effect of a higher EDE-global did not contribute to longer time to successful completion but was reversed and reduced.

Further, the hazard ratio of baseline relative BMI was 10.35, which corresponds to an increase in risk (in this context chance of successful treatment completion) of 26% for a zero-point-one increase in baseline relative BMI, i.e., 0.8 vs. 0.9 (10.35^0.1 = 1.26). Since the relative BMI values in our population takes values in a numerically small range, any noticeable difference to the risk requires a high HR coefficient.

#### Non-AN-specific factors

Several non-AN factors were also significant for time to successful treatment completion: Comorbidity of autism or a behavioural or emotional disorder, practical, economic, or health-related adversities in the family, and mental health issues in child prior to AN debut (Table 6).

A comorbid diagnosis of autism (*N* = 136) was associated with a median treatment time of 17.6 months compared to 11.6 months for those without, and 66% risk of still being in treatment after 12 months. However, a behavioural or emotional disorder (*N* = 49) implied a longer median treatment time of 24.7 months compared to 11.8 months for those without, and 76% risk of still being in treatment after 12 months.

Practical, economic, or health-related adversities in the family (*N* = 39) were associated with a median treatment time of 18 months compared to 12.2 months for those without, and 67% risk of still being in treatment after 12 months compared to 50% for those without. And participants, whose parents indicated mental health issues in child prior to AN debut (*N* = 78) had a median treatment time of 17.8 months and 66% chance of still being in treatment by 12 months, compared to 11.8 months and 49% for those without.

### Analysis 2b: additional predictors of successful treatment by 4 weeks

Analysis 2b analyzed whether variables observed at the 4-week visit provided additional insights into the time to successful treatment completion. There was information by 4 weeks for *N* = 581 participants. Number of participants is lower than analysis of start data, as 16 participants ended treatment at or before 4 weeks, 16 participants were still in day program, 37 was in inpatient treatment, and for 4 it was not possible to obtain status-information for other reasons than the above, and these participants were omitted from analysis 2b. Of the 581 participants with 4-week information, six did not receive classical FBT during the first month, due to either need for intensified treatment in part of the first month, or that a non-classical approach was chosen for clinical reasons (variations of individual therapy or half of session time with YP and half with parents or whole family). Data from these six individuals were kept in analyses. Significance and effect sizes of all the individually tested variables can be found in Online Resource 4.

The 4-week relative BMI was more significant than the baseline relative BMI, and baseline BMI lost significance, indicating that the 4-week relative BMI is more indicative of the time to successful treatment completion compared to baseline relative BMI.

The effect of EDE-global score, and the effect of practical, economic, or health-related adversities in the family both lost significance when 4-week variables were added to the model indicating that 4-week information had a stronger effect on outcome than EDE-global score, and the effect of practical, economic, or health-related adversities in the family. In contrast, mental health issues in child prior to AN debut retained a significant effect when 4-week information was taken into account.

Several 4-week variables increased the time to successful treatment completion and added significantly to the final model of analysis 2a (Table 7). These were: Compulsive exercise, higher levels of feeling fat, therapist assessment that a) YP was not able to take co-responsibility for working against AN, b) parents were less able to help YP through difficult emotions, c) it was difficult for parents to take a leading role in renourishment of YP, and d) there was other treatment challenges (e.g.; diverging views on illness, personality traits, a parent absent, ongoing divorce or other aspects of family life interfering with treatment).

**Table 7:** Hazard ratios of successful treatment completion when variables from 4 weeks of treatment were taken into account

Covariates	level	*N* in level	*N* in level with succesful completion	*p* value	HR estimate	lower confidence interval	upper confidence interval
Type of AN	F50.1	261	155	0.006	1.40	1.10	1.78
Type of AN	F50.0	320	170	0.006	1.00		
Mental health issues in child prior to AN debut	Yes	67	21	< 0.001	0.12	0.03	0.45
Mental health issues in child prior to AN debut	No	514	304	< 0.001	1.00		
Autism spectrum (ICD-10: F80-88)	Yes	112	38	0.001	0.57	0.40	0.82
Autism spectrum (ICD-10: F80-88)	No	469	287	0.001	1.00		
Behavioral or emotional disorder (ICD-10: F90-98)	Yes	41	9	< 0.001	0.34	0.17	0.68
Behavioral or emotional disorder (ICD-10: F90-98)	No	540	316	< 0.001	1.00		
Relative BMI, start of treatment			325	< 0.001	8.78	2.68	28.80
Compulsive exercise during last 4 weeks	Yes	162	73	< 0.001	0.61	0.46	0.79
Compulsive exercise during last 4 weeks	No	419	252	< 0.001	1.00		
Feeling fat during last 4 weeks, 7-point Likert-scale			325	< 0.001	0.91	0.87	0.96
YP not able to take co-responsibility for working against AN*	Unchecked	346	173	< 0.001	0.64	0.51	0.80
YP not able to take co-responsibility for working against AN*	Checked	235	152	< 0.001	1.00		
Difficult for parents to take a leading role in renourishment of YP*	Unchecked	465	279	0.042	1.38	1.00	1.90
Difficult for parents to take a leading role in renourishment of YP*	Checked	116	46	0.042	1.00		
Parents less able to help YP through difficult emotions*	Unchecked	474	284	0.005	1.59	1.13	2.24
Parents less able to help YP through difficult emotions*	Checked	107	41	0.005	1.00		
Other treatment challenges*	Unchecked	536	297	0.004	0.53	0.36	0.79
Other treatment challenges*	Checked	45	28	0.004	1.00		
Interaction effect between EDE global score and mental health issues in child prior to AN debut	No mental health issues	575	328	0.006	reference		
Interaction effect between EDE global score and mental health issues in child prior to AN debut	Yes to mental health issues	67	21	0.006	1.56	1.09	2.23

### Analysis 3: predictors for need for intensified treatment

The objective of analysis 3 was to assess which variables affected the risk of participants being admitted to intensified treatment (day program and/or inpatient hospitalization) during their course of AN treatment. Significance and effect sizes of all the individually tested variables can be found in Online Resource 5. The analysis population was the same as for analysis 2a. A total of 143 (22%) participants had one or more period of intensified treatment.

The final model estimated that atypical AN (F50.1) (atypical with regards to a smaller weight loss), baseline relative BMI, EDE-global, coexisting autism, comorbid depression, and clinician´s assessment of mother’s ability to take an active role in renourishment, all significantly influenced the risk of need for intensified treatment (Table 8).

**Table 8:** Adjusted probabilities of intensified treatment in each group of each covariate

Covariates	Group	N in level	N in level with intensified treatment	*p*-value	Adjusted probability	Adjusted probability, lower CI limit	Adjusted probability, upper CI limit	OR	OR lower CI limit	OR upper CI limit
Atypical AN (ICD-10: F50.1) due to a smaller weight loss	*No*	*526*	*132*	*0.005*	*0.21*	*0.17*	*0.25*	*Reference*		
Atypical AN (ICD-10: F50.1) due to a smaller weight loss	*Yes*	*127*	*11*	*0.005*	*0.09*	*0.05*	*0.16*	*0.38*	*0.18*	*0.75*
Mother’s ability to take an active role in renourishment*	*0*	*462*	*92*	*0.009*	*0.16*	*0.13*	*0.20*	*1.51*	*1.11*	*2.04*
Mother’s ability to take an active role in renourishment*	*1*	*144*	*32*	*0.009*	*0.22*	*0.18*	*0.27*			
Mother’s ability to take an active role in renourishment*	*2*	*46*	*19*	*0.009*	*0.30*	*0.20*	*0.42*			
EDE global score	*3*		*143*	*0.001*	*0.18*	*0.15*	*0.21*			
EDE global score	*4*			*0.001*	*0.22*	*0.18*	*0.26*	*1.28*	*1.10*	*1.50*
Autism spectrum (ICD-10: F80-88)	*No*	*517*	*88*	< *0.001*	*0.15*	*0.12*	*0.19*			
Autism spectrum (ICD-10: F80-88)	*Yes*	*136*	*55*	< *0.001*	*0.32*	*0.24*	*0.41*	*Reference*		
Affective disorders (ICD-10: F30-38)	*No*	*619*	*127*	*0.032*	*0.17*	*0.14*	*0.21*	*2.58*	*1.63*	*4.09*
Affective disorders (ICD-10: F30-38)	*Yes*	*34*	*16*	*0.032*	*0.34*	*0.19*	*0.53*	*Reference*		
Relative BMI, start of treatment	*0.8*		*143*	< *0.001*	*0.22*	*0.18*	*0.26*	*2.47*	*1.08*	*5.65*
Relative BMI, start of treatment	*0.9*			< *0.001*	*0.14*	*0.11*	*0.18*	*0.004*	*0.00*	*0.04*

Legend: *N* Number; *p* Significance level; *OR* Odds ratio; *CI* Confidence interval; *AN* Anorexia nervosa, ICD-10 = WHO´s International Classification of Diseases, 10 th edition, F50.1 = atypical anorexia nervosa, EDE = Eating Disorder Examination, global EDE = global score

**Fig. 3:**
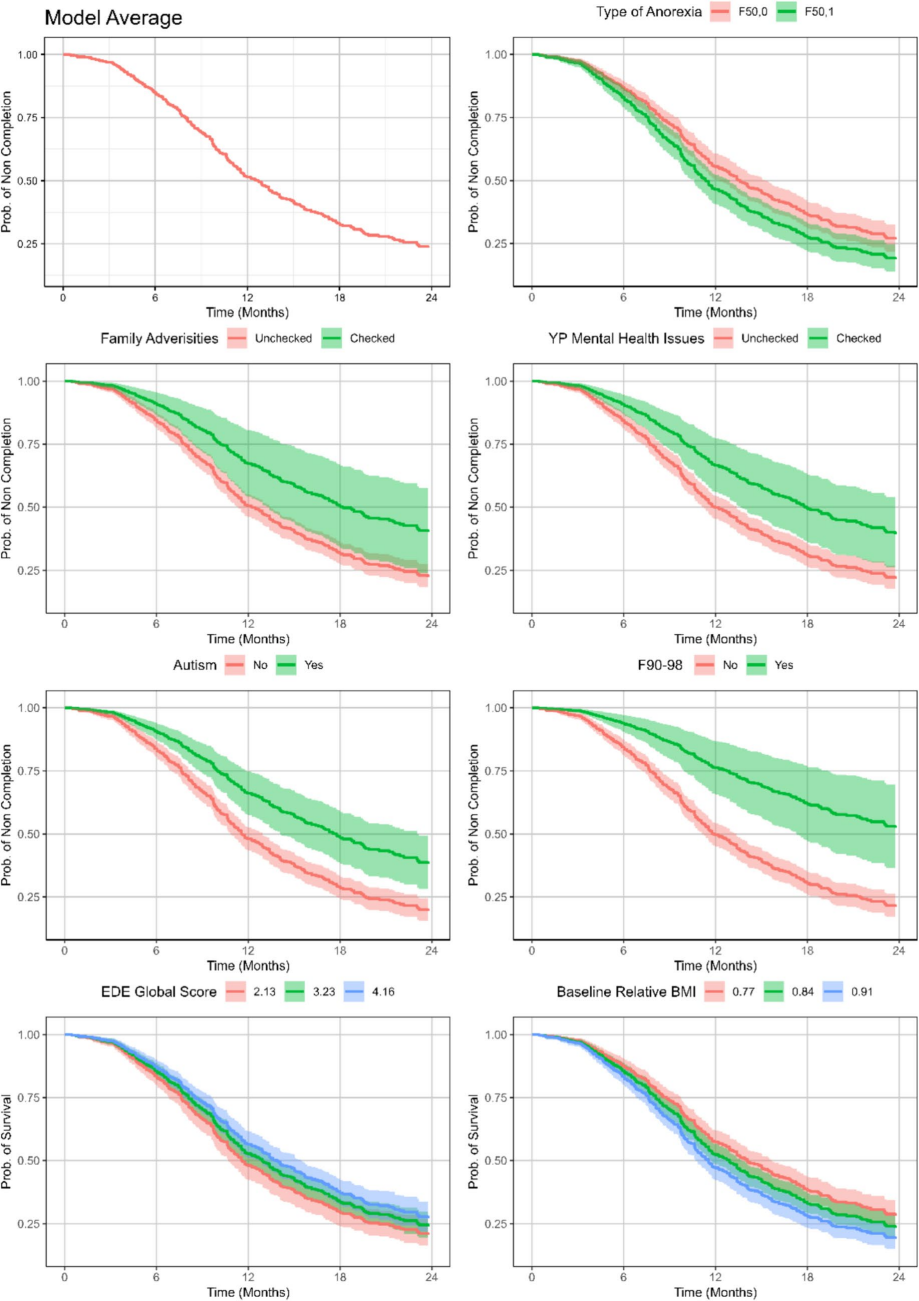
Analysis of time to successful treatment completion (analysis 2a): Adjusted survival curves from each group of each covariate. Note: Adjusted survival curves represent survival probabilities that have been averaged over the distribution of covariates in a Cox proportional hazards model. The curves give a general view by accounting for the effects of all covariates in the model. The adjustment allows for comparing survival across groups while controlling for the influence of other variables. For the numerical covariates EDE global score and relative BMI plots show the survival curves at the 25%, 50% and 75% quantiles of the covariates, since there are no groups available

**Fig. 4:**
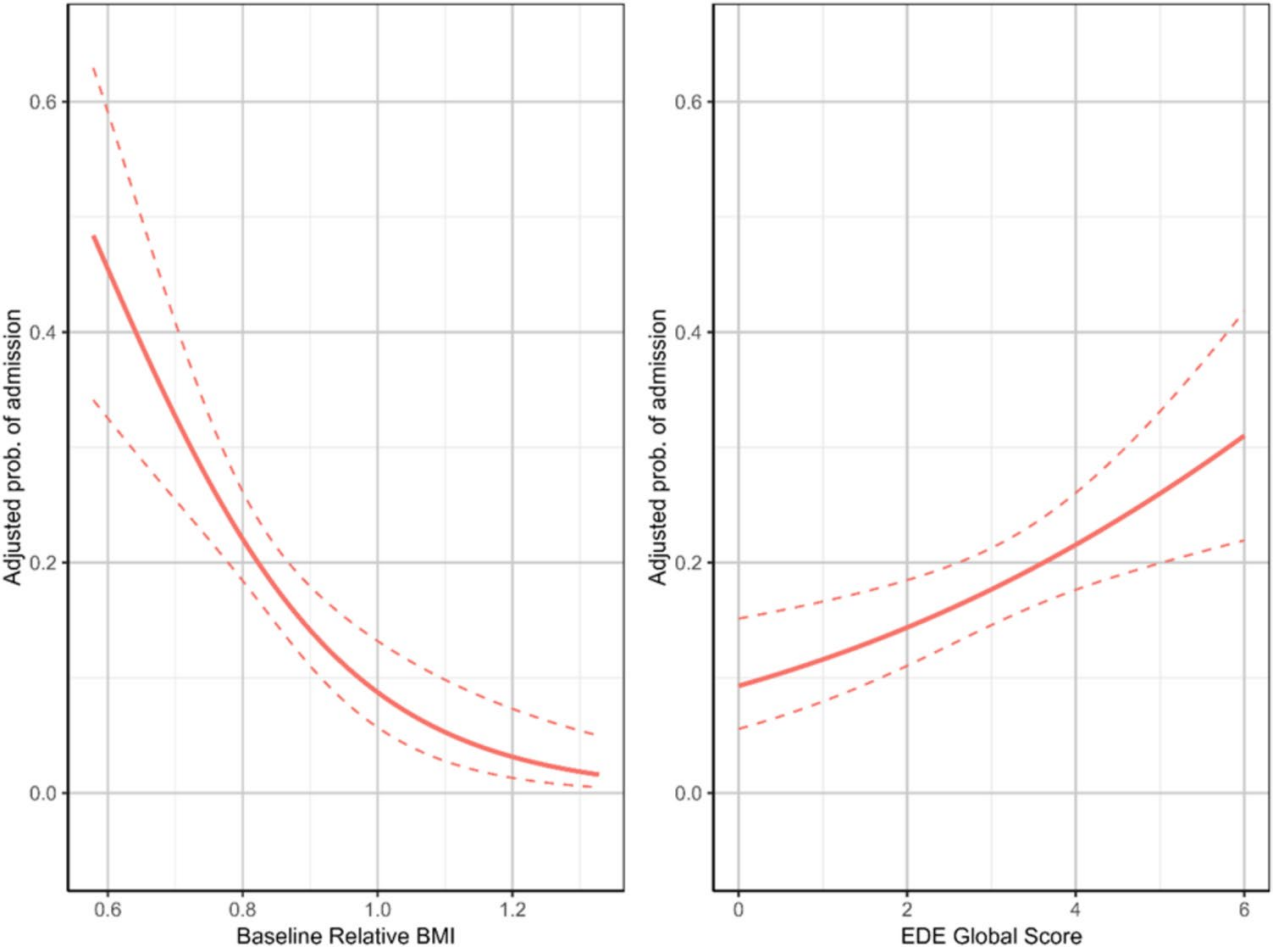
Analysis of intensified treatment (analysis 3). Adjusted probability of intensified treatment as a function of the continuous covariates baseline relative BMI and EDE global score

Participants with atypical AN due to a smaller weight loss had 9% risk of intensified treatment as opposed to a risk of 21% for the remaining participants (i.e., typical AN or atypical AN for other reasons than weight loss) when all other significant variables are controlled for. Participants with a higher relative BMI at intake had lower probability of intensified treatment: 14% if intake weight was 0.9 of median BMI for age and sex, but 22% if intake weight was only 0.8 of median BMI for age and sex at baseline (Fig. 3 and 4). Moreover, a higher EDE-global was associated with higher probability of intensification: Whereas an EDE-global of 3 implied a 18% probability, an EDE-global of 4 implied a 22% probability (Fig. 3 and 4). Autism involved a 32% probability of intensification compared to 15% for those without autism. Participants with depression had 34% probability of intensification as opposed to a 17% probability for those without. Regarding the clinician´s assessment of mother´s capacity to take on an active role in renourishment, the probability rose from 16% if capacity was assessed to be present, to 22% when uncertain, and 30% when challenged (Fig. 3).

## Discussion

This study analyzed a large sample of YPs with AN to improve knowledge of which patients have a sub-optimal response to FBT. We examined predictors for three different aspects of treatment response: Weight gain trajectory, time to successful treatment completion, and the risk of need for intensified treatment (day program or inpatient stay). A broad range of factors related to AN, to the YP, and to the family were assessed. When tested together, a few factors appeared to overrule others that seemed significant when tested separately.

First, we assessed predictors of weight gain trajectories (analysis 1). The weight trajectories revealed a clear pattern: the largest weight increase occurred within the first 3 months of FBT with slower gains later. This corroborates earlier findings and is aligned with the treatment goal of prioritizing renourishment first (J. [29]). Surprisingly, only a few additional factors influenced weight gain: Comorbid diagnosis of behavioural or emotional disorder and older age at intake was associated with less weight gain, while a history of bullying was associated with more. Unlike Lebow et al. [26], we did not identify distinct weight gain trajectories.

Second, we assessed predictors of time to successful treatment completion at baseline and after 4 weeks (analysis 2a & 2b). YPs with typical AN, higher EDE-global, or lower baseline relative BMI took longer to successful treatment completion. The same applied for YPs with family adversities, pre-existing mental health issues, autism, or behavioural or emotional disorder. In addition, relative BMI after 4 weeks was a stronger predictor of time to successful treatment completion than baseline relative BMI. Additionally, compulsive exercise, higher levels of feeling fat, and several therapist-rated moderating factors at 4 weeks also predicted longer treatment duration.

Third, we examined predictors of intensified treatment (analysis 3). A lower baseline BMI, higher EDE-global, autism, depression, and clinician´s assessment of mother’s capacity to actively support renourishment were associated with an elevated risk of need for intensified treatment.

### The role of initial underweight

The degree of initial underweight, and in extension pre-treatment weight loss, was an important predictor: it predicted longer time to successful treatment completion (analysis 2a) and a higher risk of need for intensified treatment (analysis 3). This highlights the importance of early detection and easy access to intervention in YPs showing weight loss. Delays leading to larger weight loss may prolong treatment, potentially worsen prognosis, and increase the need for day- og inpatient care, posing risk and raising health care costs. While unknown factors that caused the pretreatment weight loss may also influence treatment response, lower relative BMI was consistently linked to longer treatment and more frequent intensification even when accounting for other moderators. This reinforces prior calls for early detection and intervention [48]. Efforts like the *First Episode and Rapid Early Intervention service for Eating Disorders* (FREED) approach [13, 36], family-based intervention for high-risk youth [32], and Family-Based Treatment for Primary Care (FBT-PC) [25], aim to put this knowledge into practice.

### The role of weight gain in first month of treatment

The weight gained in the first month of treatment appears crucial, as 4-week relative BMI was a stronger predictor of time to successful treatment completion than initial relative BMI. This aligns with earlier findings that early weight gain predicts remission by end of treatment [10, 24, 35]. These insights have inspired an additional intervention focusing on improving meal support in case of insufficient weight gain in the first month [28]. Our study adds that other mental health challenges in YP in general, and specifically autism and behavioural or emotional disorder affect treatment duration beyond early weight gain. The study is not able to map out the processes by which comorbid symptoms and disorders interferes with the parental endeavors to secure renourishment, and further studies are needed so these processes may be systematically targeted in treatment. One hypothesis is that a higher need for predictability and stronger emotional reactions in some YP may be challenging to contain for families. The finding highlights the need for early screening and individualized interventions that address additional mental health challenges in YP, the additional demands they place on the parental task, and the impact they have on renourishment and regaining eating autonomy.

### The role of psychological distress

The finding that a higher baseline EDE-global increased the risk of treatment intensification suggests that, although FBT places responsibility for renourishment on the parents, thus bypassing the YP’s fluctuating motivation, the severity of YPs’ psychological distress still affects treatment outcomes, raising the likelihood of intensification. More severe levels of feeling fat, and YP being less able to take co-responsibility for working against AN as rated by week 4, seem to represent similarly high level of eating-disorder distress, and affected time to successful treatment completion. Introducing targeted interventions for YPs with greater psychological distress, addressing their cognitive and emotional challenges alongside the physical aspects of AN might improve outcome. Similarly, if clinicians by week 4 assessed that parents were less able to help patient through difficult emotions, this predicted longer time to treatment completion. Managing emotional distress appear to be a crucial skill for both YPs and parents to succeed in FBT, alongside the necessary behavioural change. Indeed, evaluating and supporting such skills may easily be overlooked due to the initial focus on somatic danger and the behavioural focus of FBT, but qualitative evidence suggests it could strengthen FBT [54]. For instance, emotion-focused strategies [9, 23, 42] and dialectical behavioural therapy strategies [18, 43] may integrate effectively with FBT.

### The role of age

Higher age was linked to less weight gain (analysis 1), but not to time to treatment completion (analysis 2). Other studies also show that older YPs with AN tend to have poorer outcomes from FBT and other treatments [11, 30], thus it is surprising that age only predicted lower weight gain. Given that insufficient weight normalisation is a known risk factor for lower remission rates in adults with AN [5, 38], our findings suggest that older adolescents’ weight trajectories need specific clinical focus. In phase one of FBT, weight restoration relies heavily on parents’ authority, but studies point to it being harder to reassert parental control in older YPs, due to their pre-AN independence. Authors recommend negotiating collaboration and co-influence when treating older YPs with FBT [8, 37]. This pinpoints a clinical dilemma: balancing between securing therapeutic alliance and taking YPs perspective into account, while ensuring sufficient weight gain to protect against long-term risks.

### The role of comorbidity

A diagnosis of behavioural or emotional disorder (ICD-10: F90-98) was linked to both less weight gain (analysis 1) and prolonged time to successful treatment termination (analysis 2). In our sample, 7,5% had comorbid F90-98, such as ADHD, supporting prior studies showing higher prevalence of attention-deficit disorders among people with AN [4, 45]. Moreover, ADHD and AN may together worsen mental well-being [2]. Thus, it is unsurprising that behavioural emotional disorders negatively impacted treatment response in this study, calling for specific attention during FBT. However, the small number of YPs with this comorbidity should be noted.

Autism was more common than behavioural or emotional disorders in our sample; 21% had or received an autism diagnosis. Frequency in other AN studies differ between 4 and 31%, perhaps due to different sampling and assessment procedures [20, 51]. A diagnosis of autism in the current study predicted longer time to successful treatment termination and increased risk of intensification, consistent with research showing lower success rates in this population [44]. While necessary, intensified treatment may present risks, for instance as the structured inpatient environment can foster dependence and prolonged hospitalization [22]. On the other hand, if treatment is prolonged because outpatient treatment is not sufficient it may carry a risk of chronification. Balancing when to intensify or continue slower FBT progress is challenging, but we suggest continuing outpatient FBT if gradual progress is evident, while accommodating the YP’s autism-related needs. Autism-friendly adaptations, such as predictability, reduced social-communicative demands, and accommodating sensory challenges, might improve FBT [33] with further guidance offered by the PEACE pathway [19, 46].

Comorbid depression was associated with a higher risk of intensification. While depressive symptoms are a frequent complication of AN, a diagnosis of depression should be assigned only when the depressed mood is beyond what is inherent in AN and/or was present before or persists despite weight normalisation. Our finding suggests that comorbid depression can interfere with treatment and should not be overlooked. An integrated approach targeting both the eating disorder and affective disorder may help prevent intensification.

### The role of family factors

No aspects of family adversities or carer status assessed in this study influenced weight gain trajectory. This is noteworthy since carer burden and parental resources naturally have been in focus in FBT literature, as parents are the primary agents of change. While family factors did not impact on weight gain, they impacted on time to successful treatment completion: Hence practical, economic, or health-related adversities, therapist assessment by week 4 that it was difficult for parents to take a leading role in renourishment of YP, or that parents were less able to help YP through difficult emotions, all significantly influenced the time to successful treatment completion. Evidence on family factors in FBT is mixed; an earlier study found that families with separated parents in separate households required longer time in FBT [27], while another study found no significant impact of family structure, income, YP comorbidity, or parents'self-efficacy at baseline on FBT outcome [7]. By week 4 of the study, family adversities lost significance, likely because other variables were stronger. The therapist´s assessment that it was difficult for parents to take a leading role in renourishment of YP, that parents struggled to help YP through difficult emotions and that other treatment challenges existed might better capture how family adversities impact on FBT tasks. Overall, our findings suggest that the strong momentum in early FBT and multidisciplinary team support help families even with additional challenges to take an active stance and initiate weight restoration. However, treatment may be prolonged if other adversities strain family resources, especially if this manifests itself in reduced capacity for taking on a leading role or containing strong emotions. It should be noted, however, that several assessments, including the assessment of parental capacity for taking on a leading role or containing strong emotions, was based on a simple clinician evaluation, and that a comprehensive and validated instrument assessing these factors might give more valid information.

### The role of clinician-rated assessments

Several clinician-ratings turned out as significant predictive factors; mother´s capacity to take on an active role in renourishment assessed by intake, YP´s reduced ability to take co-responsibility for working against AN, difficulty for parents to take a leading role in renourishment, and parents´ reduced ability to help YP through difficult emotions assessed by 4 weeks were all significant. While these were generic Likert or dichotomous ratings, not validated scales, they highlight the importance of clinicians´ consideration of factors that may affect therapy. This study suggests that treatment team´s reflections on moderating factors in FBT contain valuable predictive insight and should impact treatment planning in clinical practice, although it would be preferable to supplement by independent assessments to evaluate the effect of bias.

### Other findings

Mothers´ but not fathers´ capacity played a role in the risk of intensification. FBT specifically focus on parental alignment and on involvement of both parents. Despite Denmark's focus on gender equality, the lack of a similar association with the father’s ability to take an active role in renourishment likely reflects the fact that it is still primarily mothers who take leave of absence from work and assume the day-to-day responsibility for the refeeding process.

It was surprising that a history of bullying was associated with greater weight gain. This finding should be confirmed in other samples to rule out random findings (type 2 error). A speculative explanation for the role of prior bullying might be that the YPs felt relieved by the break from school and responded well to the time at home with a parent, free from peer-related adversities during renourishment.

### Strengths

The hospital unit where the study took place, covers the largest region of Denmark and is the only publicly funded, hospital-based service for youth with eating disorders in the region. The primary strength of this study is its large, geographically representative sample that was monitored prospectively.

### Limitations

Main limitations stem from the study being a case series from a single site with no comparison to other treatments or usual care and limited fidelity measures. A further limitation is missing data at varied time points, particularly at the end of treatment. Although unfortunate, this is a common issue in clinical psychiatric cohorts [40]. Moreover, several variables, including the assessment of parental capacity for taking on a leading role or containing strong emotions, and assessment of motivation, was based on a simple and generic clinician evaluation. Comprehensive and validated instruments assessing these factors would give better information and are needed in future studies. Also, assessment of clinical presentation and outcomes were performed by internal clinicians, and not independent researchers. Lastly, this was a naturalistic study with no formal treatment fidelity measures, and with adaptations to manualized FBT, especially regarding no fixed duration, and therefore conclusions may not be fully generalizable to FBT delivery within 20 sessions and with control of adherence to manualized FBT.

## Conclusions

Our analyses identified several characteristics of YPs who do not sufficiently benefit from outpatient FBT in standard care. These include lower relative BMI og high level of psychological AN-symptoms at intake, and even more importantly, relative BMI after the first month of treatment. Other risk factors include prior mental health issues and comorbid psychiatric conditions in YP, as does YPs´ lower ability to take a co-responsibility for treatment progress, and parents struggling to support their YP through emotional reactions. This at-risk profile calls for early clinical attention, screening and developing strategies to strengthen FBT for YPs with these profiles, potentially improving recovery rates and reducing the need for more invasive interventions.

## Supplementary Information

Below is the link to the electronic supplementary material.

Supplementary file1 (PDF 426 KB)

Supplementary file2 (PDF 446 KB)

Supplementary file3 (PDF 443 KB)

Supplementary file4 (PDF 453 KB)

Supplementary file5 (PDF 460 KB)

## Data Availability

The data that support the findings of this study may be made available from the corresponding author upon reasonable request. However, restrictions may apply depending on whether data can be sufficiently anonymized at the time of request and may require specific permission from The Danish Data protection Agency.

## Declarations

### Clinical trial registration

The study is registered in the ClinicalTrials.gov research database (ClinicalTrials.gov Identifier: NCT05956366).
