# Supplementary material for: Predictors of response to family-based treatment for anorexia nervosa in youth: insights from the VIBUS project
Source: Eur Child Adolesc Psychiatry. 2025 Jun 11;34(11):3665–84. doi: 10.1007/s00787-025-02766-x (PMC12647301; doi:10.1007/s00787-025-02766-x)
Supplement: Supplementary file 1 — Supplementary file1 (PDF 426 KB) [file 787_2025_2766_MOESM1_ESM.pdf]

Online Resource 1 for the manuscript:

Family Based Treatment for anorexia nervosa: Trajectories of improvement and characteristics of those who do not benefit sufficiently - A longitudinal study

European Child & Adolescent Psychiatry

Mette Bentz, Signe Holm Pedersen, Ulla Moslet, Nikolaj Petersen, Anne Katrine Pagsberg

Correspondence: mette.bentz(at)regionh.dk, Child and Adolescent Mental Health Centre, Mental Health Services in the Capital Region of Denmark, Bispebjerg Bakke 30, DK 2400 Copenhagen NV

STATUS CHART (progress monitoring) - Section B132

/rev. 16.05.24

**Date:**	**Completed by (initials):**	**Patient's age:**	year	months
**Patient's name:**		**Social security number:**		
**Psychopharmacological treatment: No yes** ☐ ☐	If yes – preparation(s)?	If yes – date of last medical check:<br>*NOTE: Need for new appointment every 3 months*		
**Current action diagnosis (diagnosis code):**	**Other psychiatric diagnoses by status (diagnosis code):**	**Any somatic diagnoses by status (diagnosis code or name):**		

	**Status at (tick):**
	4 weeks (FBT 5)
	3 months
	6 months
	9 months
	12 months<br>*(REMEMBER: new treatment plan + CAVE)*
	Every 3 months thereafter (write number of months)
	When transferring from day/24-hour treatment
	At the end of treatment

	Number of therapy sessions (including this one) since the start of treatment (not including day program /inpatient stays)

	**Processing since last status** (check more than one if necessary)
	FBT
	FBT with meal group
	Day program
	Inpatient treatment
	Individual therapy – number of sessions visited:
	Extended weighing time (approx. ½ of the time with pt. alone)
	Alternating individual and family conversations
	ARFID treatment
	Parent-only sessions
	FFT (parent-focused therapy)
	Municipal contact (e.g. NVM, notification)
	Psychosocial treatment track

**1. Somatic status**		
Optional: Weight at start:	Height in cm (Measure height at each stage except 4 weeks)	
	Current weight (in kg.):	
Healthy weight curve is estimated at around (on height/weight curve)	median/+/-SD	
Healthy weight curve is estimated at around (on BMI curve)	median/+/-SD	
Healthy weight curve at current age and height, weight approx. in kg	kg.	

Questions regarding motivation and collaboration

	**2. The patient's insight into the disease**
	Poor
	Limited/little
	Some/moderate
	Good

	**3. The patient's motivation for change**
	Poor
	Ambivalent/predominantly negative
	Ambivalent/mostly positive
	Motivated, acts on the desire for change

	**4a. Collaborative relationship with the patient**
	Poor
	Mixed, mostly negative
	Mixed, mostly positive
	Good

	**4b. Collaborative relationship with parents**
	Poor
	Mixed, mostly negative
	Mixed, mostly positive
	Good

Questions related to family therapy (tick the most important ones)

6a. Factors that promote recovery	
	Parents' leading role in re-nutrition
	Parents' leading role in curbing eating disordered behavior
	Parents' collaboration as a team
	Parents help through difficult emotions
	Patient is receptive to support from parents
	Patient can take responsibility for opposing SF
	Other – which one?

6c. Only for ARFID cases	
	Number of foods incorporated. *These can be either new or previously excluded foods; they count as "incorporated" if they can be eaten about once a week in a normal portion.*

7. Plan (tick) (to be completed after team conference)	
	Continue FBT – specify phase:
	FBT w/add-on (FBT5+ and FBT6+)
	Refer to the day program
	Referred to inpatient treatment
	Individual therapy
	Further assessment
	Other - specify what:
	Completed – if completed, proceed to question 8

5. About the family (tick a box)	Yes	No
Parents are cohabiting		
Both parents generally participate in therapy.		
Siblings generally participate in therapy.		

6b. Factors that hinder recovery	
	Difficult for parents to take a leading role
	Difficult for parents to help patient through difficult emotions
	Difficult for parents to cooperate
	Difficult for the patient to take responsibility for going against SF
	The family has other challenges (social, health, etc.)
	Patient´s diagnosed comorbidity
	Other psychiatric symptoms in the patient
	Patient´s problems with handling emotions/flexibility/perfectionism
	Problems with school/peer relationships
	Challenges regarding the relationship between parents and child (e.g. difficulty separating the patient from the illness / criticism / blame of the child / conflicts / resistance to / rejection of parents)
	Doubt or disagreement about the disease/treatment approach/healthy weight etc.
	Other: which one?

8. Termination method (please tick)	
	Succesfully completed treatment
	Referred to adult psychiatry (eating disorder)
	Referred to adult psychiatry (not eating disorder)
	Transferred to treatment elsewhere in BUC (other disorders)
	Continues treatment in private sector (e.g. Askovhus)
	Continues treatment under municipal administration
	Not successfully completed, stops at the family's request

8a. Note to the practitioner if you only complete pages 1-2:
– write the number of menstruations in the last three expected menstrual cycles:

8b. Is the patient taking birth control pills or other hormonal contraception?	
	Yes
	No

8c. If no menstruation, what is the reason?	
	No known cause other than low weight
	Haven't had her first period
	Boy
	Other reasons for not having a period (pregnancy, somatic illness, etc.)

Questions 8-16 (excluding 13c) **constitute diagnostic questions from the Eating Disorder Examination (EDE)**

**8a. Number of menstrual periods in the last three expected menstrual cycles**	
**8b. Are you taking birth control pills or other hormonal contraception?**	
	Yes
	No
**8c. If no menstruation, what is the reason?**	
	No known cause other than low weight
	Haven't had first period
	Boy
	Other reasons for not having a period (pregnancy, somatic illness, etc.)

**9. Have you/your child had a desire to limit eating in the last 4 weeks?** - I.e. try to limit/cut down/avoid food or would want to do so if the parents did not prevent it)? (tick)	
	No wish
	Desire for limitation on <half of the days
	Desire for limitation on half of the days
	Desire for limitation on >half of the days
	Desire for restriction on all days

**10a. Have you/your child tried to lose weight in the last 4 weeks? (tick)**	
	No, not at all
	Tried to lose weight because of figure/weight
	Tried to lose weight for other reasons

**11. Have you/your child experienced objective binge eating episodes with perceived loss of control? (tick)**	
	No, not at all
	Yes - specify number of times in the last 4 [illegible]

**10b. If the child is underweight** **Have you/your child tried to maintain a low weight (i.e. tried to avoid gaining weight) during the last 4 weeks? (tick)**	
	Not applicable (i.e. not underweight)
	No, not at all
	Tried to maintain underweight due to figure/weight)
	Tried to maintain underweight for other reasons (please note which ones):

12. Over the past four weeks, have you/your child abstained from food in any way to regulate weight and/or shape (e.g. through vomiting, medication of any kind, or other)?

	Vomiting	Laxatives	Diuretics
No			
Yes - number of times in the last 4 weeks:			

13a+b. Have you/your child engaged in compulsive exercise (to regulate weight and/or figure) in the last four weeks? (tick)

	No, not at all	*Help questions:* What happens if you can't exercise for some reason? Does it cause feelings of guilt, stress, etc.?
	Yes – number of days in the last four weeks	

13c. Eating responsibility
Who is currently responsible for adequate and regular eating? (tick)

	Parents have full responsibility for eating
	Patient has growing responsibility for eating in limited areas
	Patient has co-responsibility or is practicing increasing responsibility with support from parents
	patient has main responsibility for eating (corresponding to the normal for his/her age)
Other – what:	

14. How important is your shape to how you have felt about yourself in the last 4 weeks? (tick)		
0		No importance
1		
2		Some importance
3		
4		Moderate importance
5		
6		Supreme importance

15. How important has your weight been to how you have felt about yourself in the last 4 weeks?(tick) *		
0		No importance
1		
2		Some importance
3		
4		Moderate importance
5		
6		Supreme importance

16. Have you felt fat in the last 4 weeks? (tick)		
0		Haven't felt fat
1		
2		Have felt fat <half the days
3		
4		Have felt fat >half the days
5		
6		Have felt fat every day

*For patients who do not know their weight, say, for example: "Even if you do not know your weight, you may be able to speculate a lot about it. If you knew your weight, how much of an impact do you think it would have on how you have felt about yourself in the last 4 weeks?"

18. Assessment of quality of life. How satisfied have you been with your life in the last 4 weeks? (tick)		
1		Very satisfied
2		Satisfied
3		Neither or
4		Dissatisfied
5		Very dissatisfied

17. Current social life (check multiple answers if necessary)	
	In school/work full time
	In school/work part-time
	Not at school/work
	Seeing friends outside of school hours
	Has leisure activity(ies) outside of school hours
	A parent on leave due to SF – part-time
	A parent on leave due to SF – full-time

6c. Factors regarding treatment that may hinder or promote recovery (please tick)<br>Here you only need to tick, but if there are any wishes/challenges: describe in the status note	
	The family has no wishes for changes to the current treatment.
	The family has requests for changes (describe what in the status note)
	There are challenges regarding the framework for treatment (e.g. attendance/cancellation/no-show/change of therapist)
	There are challenges in terms of collaboration, as the therapist(s) and family have different views on… (describe in status note)
	**NOTE to therapist: Consider whether family/patient is a candidate for either**:<br>• F-ACT (if serious, needs closer follow-up)<br>• Meal group (when there are challenges with meals or weight gain)

**ATTENTION to the therapist:**
**If there have been previous examples of self-harming behavior – consider whether screening is necessary? Referred to the Self-Harm Team?**
Have you intentionally harmed yourself?
• *If yes: How many times in the last 4 weeks?*
• *If yes: How does it help you?*
