# Supplementary material for: Predictors of response to family-based treatment for anorexia nervosa in youth: insights from the VIBUS project
Source: Eur Child Adolesc Psychiatry. 2025 Jun 11;34(11):3665–84. doi: 10.1007/s00787-025-02766-x (PMC12647301; doi:10.1007/s00787-025-02766-x)
Supplement: Supplementary file 2 — Supplementary file2 (PDF 446 KB) [file 787_2025_2766_MOESM2_ESM.pdf]

Online Resource 2 for the manuscript: **Family Based Treatment for anorexia nervosa: Trajectories of improvement and characteristics of those who do not benefit sufficiently - A longitudinal study**

European Child & Adolescent Psychiatry

Mette Bentz, Signe Holm Pedersen, Ulla Moslet, Nikolaj Petersen, Anne Katrine Pagsberg
Correspondence: mette.bentz(at)regionh.dk, Child and Adolescent Mental Health Centre, Mental Health Services in the Capital Region of Denmark, Bispebjerg Bakke 30, DK 2400 Copenhagen NV

Supplementary Table: All variables screened individually for Analysis 1 (mixed model for repeated measures regarding weight trajectories) (comma as decimal separator)

Covariates	n in analysis	level	n in level	p-value	estimate	lower confidence interval	upper confidence interval
Sex	511	pige	476	0,31	0,01	-0,01	0,03
Sex	511	dreng	35	0,31			
Type of AN	511	F50,1	156	0,03	-0,01	-0,02	0,00
Type of AN	511	F50,0	355	0,03			
Atypical AN (ICD-10: F50.1) due to no avoidance of fattening foods	511	Unchecked	503	0,62	-0,01	-0,04	0,03
Atypical AN (ICD-10: F50.1) due to no avoidance of fattening foods	511	Checked	8	0,62			
Atypical AN (ICD-10: F50.1) due to not feeling fat	511	Unchecked	443	0,91	0,00	-0,01	0,01
Atypical AN (ICD-10: F50.1) due to not feeling fat	511	Checked	68	0,91			
Atypical AN (ICD-10: F50.1) due to no endocrine disturbances (i.e.; girls still menstruating)	511	Unchecked	432	0,02	0,02	0,00	0,03
Atypical AN (ICD-10: F50.1) due to no endocrine disturbances (i.e.; girls still menstruating)	511	Checked	79	0,02			
Atypical AN (ICD-10: F50.1) due to presence of bulimic symptoms	511	Unchecked	478	0,08	0,02	0,00	0,03
Atypical AN (ICD-10: F50.1) due to presence of bulimic symptoms	511	Checked	33	0,08			
Compulsive excercise	487	Yes	314	0,23	-0,01	-0,02	0,00
Compulsive excercise	487	No	173	0,23			
Compulsive exercise, if yes, no. of days during last 4 weeks	273			0,89	0,00	0,00	0,00
History of bullying in general	471	Nej	388	0,08	-0,01	-0,02	0,00
History of bullying in general	471	Ja	83	0,08			
Other adversities related to peer relationships prior to AN debut	511	Unchecked	380	0,07	-0,01	-0,02	0,00
Other adversities related to peer relationships prior to AN debut	511	Checked	131	0,07			
Relational challenges in the family prior to AN debut	511	Unchecked	446	0,36	-0,01	-0,02	0,01
Relational challenges in the family prior to AN debut	511	Checked	65	0,36			
Practical, economic, or health-related adversities in the family prior to AN debut	511	Unchecked	481	0,47	-0,01	-0,03	0,01
Practical, economic, or health-related adversities in the family prior to AN debut	511	Checked	30	0,47			
Larger changes in e.g., housing or school prior to AN debut	511	Unchecked	404	0,23	0,01	0,00	0,02
Larger changes in e.g., housing or school prior to AN debut	511	Checked	107	0,23			
A history of bullying prior to AN debut	511	Unchecked	492	0,32	0,01	-0,01	0,04

A history of bullying prior to AN debut	511	Checked	19	0,32			
A history of overweight prior to AN debut	511	Unchecked	481	0,00	0,03	0,01	0,05
A history of overweight prior to AN debut	511	Checked	30	0,00			
Mental health issues in child prior to AN debut	511	Unchecked	459	0,22	0,01	-0,01	0,02
Mental health issues in child prior to AN debut	511	Checked	52	0,22			
Somatic health issues in child prior to AN debut	511	Unchecked	500	0,40	0,01	-0,02	0,05
Somatic health issues in child prior to AN debut	511	Checked	11	0,40			
Loss or deaths of close others prior to AN debut	511	Unchecked	494	0,79	0,00	-0,02	0,03
Loss or deaths of close others prior to AN debut	511	Checked	17	0,79			
Other adversities prior to AN debut	511	Unchecked	357	0,96	0,00	-0,01	0,01
Other adversities prior to AN debut	511	Checked	154	0,96			
Prior interventions from municipal authorities	156	nej	119	0,76	0,00	-0,02	0,02
Prior interventions from municipal authorities	156	ja	37	0,76			
Traumas affecting the family	156	nej	147	0,99	0,00	-0,04	0,04
Traumas affecting the family	156	ja	9	0,99			
Somatic or mental health issues in siblings	160	nej	143	0,60	0,01	-0,02	0,03
Somatic or mental health issues in siblings	160	ja	17	0,60			
Somatic or mental illness in a parent to a degree deemed affecting their ability to take on an active role in renourishment	158	nej	150	0,09	0,03	0,00	0,07
Somatic or mental illness in a parent to a degree deemed affecting their ability to take on an active role in renourishment	158	ja	8	0,09			
Clinician´s assessment of mother’s ability to take an active role in renourishment	465	Uncertain	112	0,06	0,01	-0,01	0,03
Clinician´s assessment of mother’s ability to take an active role in renourishment	465	yes	318	0,06	0,02	0,00	0,04
Clinician´s assessment of mother’s ability to take an active role in renourishment	465	Challenged	34	0,06			
Clinician´s assessment of father’s ability to take an active role in renourishment	449	Uncertain	120	0,68	0,00	-0,01	0,02
Clinician´s assessment of father’s ability to take an active role in renourishment	449	yes	283	0,68	0,01	-0,01	0,02
Clinician´s assessment of father’s ability to take an active role in renourishment	449	Challenged	46	0,68			
Binging and/or purging behaviours	502	No	394	0,01	0,05	0,02	0,07
Binging and/or purging behaviours	502	Only binging	40	0,01	0,05	0,02	0,08
Binging and/or purging behaviours	502	Only purging	53	0,01	0,04	0,01	0,07
Binging and/or purging behaviours	502	Binging and purging	15	0,01			
EDE global score	496			0,07	0,00	-0,01	0,00
Duration of restrictive eating before start, reported by young person	376			0,67	0,00	0,00	0,00
Parents´ own assessment or their ability to take an active role in renourishment	449	In doubt	55	0,05	-0,02	-0,03	0,00
Parents´ own assessment or their ability to take an active role in renourishment	449	Yes	394	0,05			
Cargiver status	499	One responsible parent	40	0,62	-0,01	-0,03	0,01
Cargiver status	499	Other caregiver	1	0,62	0,02	-0,09	0,12
Cargiver status	499	parents living together	354	0,62	0,00	-0,02	0,01
Cargiver status	499	Living apart, both active	104	0,62			
Mental retardation or intellectualis inferioritas	511	Yes	6	0,13	-0,03	-0,08	0,01
Mental retardation or intellectualis inferioritas	511	No	505	0,13			
Autism spectrum (ICD-10: F80-88)	511	Yes	110	0,82	0,00	-0,01	0,01
Autism spectrum (ICD-10: F80-88)	511	No	401	0,82			

Behavioral or emotional disorder (F90-98)	511	Yes	34	0,00	-0,03	-0,05	-0,01
Behavioral or emotional disorder (F90-98)	511	No	477	0,00			
Affective disorders (ICD-10: F30-38)	511	Yes	22	0,62	-0,01	-0,03	0,02
Affective disorders (ICD-10: F30-38)	511	No	489	0,62			
Anxiety disorders (ICD-10: F40-48)	511	Yes	41	0,02	-0,02	-0,04	0,00
Anxiety disorders (ICD-10: F40-48)	511	No	470	0,02			
Other comorbidities	511	Yes	10	0,94	0,00	-0,03	0,03
Other comorbidities	511	No	501	0,94			
relative BMI, start of treatment	511			0,00	-0,19	-0,24	-0,15
Age at start, continous	511			0,00	-0,01	-0,01	0,00

Legend:N=number, p= significance level,OR=odds ratio, CI= confidence Interval, AN= anorexia nervosa, ICD-10=WHO´s International classification of Diseases, 10th edition, F50.1= atypical anorexia nervosa, EDE= Eating Disorder Examination, global EDE= global score of psychological symptoms derived from the EDE, BMI=Body mass index , relative BMI= actual BMI divided by the population-based median BMI for sex and age, *=assesment by therapist
