# Supplementary material for: Predictors of response to family-based treatment for anorexia nervosa in youth: insights from the VIBUS project
Source: Eur Child Adolesc Psychiatry. 2025 Jun 11;34(11):3665–84. doi: 10.1007/s00787-025-02766-x (PMC12647301; doi:10.1007/s00787-025-02766-x)
Supplement: Supplementary file 3 — Supplementary file3 (PDF 443 KB) [file 787_2025_2766_MOESM3_ESM.pdf]

Online Resource 3 for the manuscript: **Family Based Treatment for anorexia nervosa: Trajectories of improvement and characteristics of those who do not benefit sufficiently - A longitudinal study**

European Child & Adolescent Psychiatry

Mette Bentz, Signe Holm Pedersen, Ulla Moslet, Nikolaj Petersen, Anne Katrine Pagsberg
Correspondence: mette.bentz(at)regionh.dk, Child and Adolescent Mental Health Centre, Mental Health Services in the Capital Region of Denmark, Bispebjerg Bakke 30, DK 2400 Copenhagen NV

Supplementary Table: Significance and effect sizes of all the individually tested variables in analysis 2a (baseline predictors of time to successful treatment completion) (comma as decimal separator)

Covariates	N in analysis	level	N in level	n in level with succesful completion	p-value	estimate	lower CI limit	upper CI limit
Sex	653	Female	610	322	0,09	0,70	0,48	1,03
Sex	653	Male	43	28	0,09	1,00		
Age at start, continous	653				0,92	1,00	0,94	1,08
Age at start, discrete agegroups	653	13-15	272	149	0,70	0,88	0,66	1,18
Age at start, discrete agegroups	653	15-18	264	134	0,70	0,90	0,67	1,21
Age at start, discrete agegroups	653	<13	117	67	0,70	1,00		
Compulsive excercise	618	Yes	405	215	0,03	0,78	0,62	0,97
Compulsive excercise	618	No	213	118	0,03	1,00		
Compulsive exercise, if yes, no. of days during last 4 weeks	356				0,79	1,00	0,99	1,02
Compulsive exercise, if yes, no. of days during last 4 weeks, discrete groups	356	0	23	14	0,42	1,25	0,72	2,19
Compulsive exercise, if yes, no. of days during last 4 weeks, discrete groups	356	1-15	131	66	0,42	0,87	0,64	1,18
Compulsive exercise, if yes, no. of days during last 4 weeks, discrete groups	356	>15	202	106	0,42	1,00		
Atypical AN (F50.1)	653	F50,1	288	162	0,00	1,50	1,22	1,86
Atypical AN (F50.1)	653	F50,0	365	188	0,00	1,00		
Atypical AN (F50.1) due to smaller weight loss	653	Unchecked	526	280	0,04	0,75	0,58	0,98
Atypical AN (F50.1) due to smaller weight loss	653	Checked	127	70	0,04	1,00		
Atypical AN (ICD-10: F50.1) due to no avoidance of fattening foods	653	Unchecked	645	347	0,76	1,19	0,38	3,71
Atypical AN (ICD-10: F50.1) due to no avoidance of fattening foods	653	Checked	8	3	0,76	1,00		
Atypical AN (ICD-10: F50.1) due to not feeling fat	653	Unchecked	579	308	0,22	0,81	0,59	1,12
Atypical AN (ICD-10: F50.1) due to not feeling fat	653	Checked	74	42	0,22	1,00		
Atypical AN (ICD-10: F50.1) due to no endocrine disturbances (i.e.; girls still menstruating)	653	Unchecked	536	283	0,00	0,67	0,51	0,88
Atypical AN (ICD-10: F50.1) due to no endocrine disturbances (i.e.; girls still menstruating)	653	Checked	117	67	0,00	1,00		
Atypical AN (ICD-10: F50.1) due to presence of bulimic symptoms	653	Unchecked	612	329	0,78	0,94	0,60	1,46
Atypical AN (ICD-10: F50.1) due to presence of bulimic symptoms	653	Checked	41	21	0,78	1,00		
History of bullying in general	602	No	487	262	0,48	0,90	0,68	1,19
History of bullying in general	602	Yes	115	62	0,48	1,00		
Other adversities related to peer relationships prior to AN debut	653	Unchecked	486	268	0,01	1,36	1,06	1,75
Other adversities related to peer relationships prior to AN debut	653	Checked	167	82	0,01	1,00		
Relational challenges in the family prior to AN debut	653	Unchecked	568	308	0,62	1,09	0,78	1,50
Relational challenges in the family prior to AN debut	653	Checked	85	42	0,62	1,00		

Practical, economic, or health-related adversities in the family prior to AN debut	653	Unchecked	614	334	0,02	1,71	1,04	2,83
Practical, economic, or health-related adversities in the family prior to AN debut	653	Checked	39	16	0,02	1,00		
Larger changes in e.g., housing or school prior to AN debut	653	Unchecked	520	288	0,08	1,27	0,96	1,67
Larger changes in e.g., housing or school prior to AN debut	653	Checked	133	62	0,08	1,00		
A history of bullying prior to AN debut	653	Unchecked	629	336	0,24	0,71	0,42	1,22
A history of bullying prior to AN debut	653	Checked	24	14	0,24	1,00		
A history of overweight prior to AN debut	653	Unchecked	619	334	0,16	0,68	0,41	1,13
A history of overweight prior to AN debut	653	Checked	34	16	0,16	1,00		
Mental health issues in child prior to AN debut	653	Unchecked	575	327	0,00	2,27	1,48	3,46
Mental health issues in child prior to AN debut	653	Checked	78	23	0,00	1,00		
Somatic health issues in child prior to AN debut	653	Unchecked	638	342	0,73	1,13	0,56	2,28
Somatic health issues in child prior to AN debut	653	Checked	15	8	0,73	1,00		
Loss or deaths of close others prior to AN debut	653	Unchecked	631	344	0,05	2,04	0,91	4,56
Loss or deaths of close others prior to AN debut	653	Checked	22	6	0,05	1,00		
Other adversities prior to AN debut	653	Unchecked	448	236	0,10	0,83	0,66	1,04
Other adversities prior to AN debut	653	Checked	205	114	0,10	1,00		
Prior interventions from municipal authorities	204	No	156	86	0,00	2,08	1,20	3,60
Prior interventions from municipal authorities	204	Yes	48	15	0,00	1,00		
Traumas affecting the family	205	No	193	98	0,10	2,13	0,78	5,86
Traumas affecting the family	205	Yes	12	4	0,10	1,00		
Somatic or mental health issues in siblings	208	No	184	94	0,12	1,67	0,84	3,32
Somatic or mental health issues in siblings	208	Yes	24	9	0,12	1,00		
Somatic or mental illness in a parent to a degree deemed affecting their ability to take on an active role in renourishment	206	Binging and purging	192	95	0,89	1,06	0,49	2,31
Somatic or mental illness in a parent to a degree deemed affecting their ability to take on an active role in renourishment	206	Yes	14	7	0,89	1,00		
Clinician´s assessment of mother’s ability to take an active role in renourishment, 3-point Likert scale	597				0,10	0,85	0,70	1,03
Clinician´s assessment of father’s ability to take an active role in renourishment, 3-point Likert scale	578				0,88	1,01	0,85	1,20
Binging and/or purging behaviours	643	No	487	262	0,91	0,85	0,48	1,52
Binging and/or purging behaviours	643	Only binging	52	28	0,91	0,86	0,44	1,70
Binging and/or purging behaviours	643	Only purging	78	45	0,91	0,93	0,49	1,76
Binging and/or purging behaviours	643	Binging and purging	26	12	0,91	1,00		
EDE global score	633				0,00	0,88	0,81	0,95
Duration of restrictive eating before start, reported by young person	483				0,76	1,00	0,99	1,02
Parents´ own assessment or their ability to take an active role in renourishment		No			0,20	0,00	0,00	Inf
Parents´ own assessment or their ability to take an active role in renourishment		In doubt	69	28	0,20	0,75	0,51	1,11
Parents´ own assessment or their ability to take an active role in renourishment		Yes	503	281	0,20	1,00		
Cargiver status	638	One responsible parent	47	23	0,46	1,09	0,68	1,75
Cargiver status	638	Other caregiver	3	2	0,46	3,26	0,80	13,34
Cargiver status	638	parents living together	461	249	0,46	0,94	0,72	1,23
Cargiver status	638	Living apart, both active	127	68	0,46	1,00		
Mental retardation or intellectualis inferioritas	653	Yes	8	3	0,39	0,63	0,20	1,96
Mental retardation or intellectualis inferioritas	653	No	645	347	0,39	1,00		
Autism spectrum (ICD-10: F80-88)	653	Yes	136	46	0,00	0,44	0,32	0,61

Autism spectrum (ICD-10: F80-88)	653	No	517	304	0,00	1,00		
Behavioral emotional disorder (ICD-10: F90-98)	653	Yes	49	12	0,00	0,34	0,19	0,61
Behavioral emotional disorder (ICD-10: F90-98)	653	No	604	338	0,00	1,00		
Affective disorders (ICD-10: F30-38)	653	Yes	34	9	0,00	0,36	0,19	0,71
Affective disorders (ICD-10: F30-38)	653	No	619	341	0,00	1,00		
Anxiety disorders (ICD-10: F40-48)	653	Yes	53	17	0,00	0,49	0,30	0,80
Anxiety disorders (ICD-10: F40-48)	653	No	600	333	0,00	1,00		
Other comorbidities	653	Yes	12	5	0,95	1,03	0,43	2,50
Other comorbidities	653	No	641	345	0,95	1,00		
relative BMI, start of treatment, continous	653				0,00	10,86	4,25	27,76
relative BMI, start of treatment, discrete groups	653	>1	51	28	0,00	1,65	1,09	2,50
relative BMI, start of treatment, discrete groups	653	0.8-1	376	211	0,00	1,45	1,15	1,83
relative BMI, start of treatment, discrete groups	653	<0.8	226	111	0,00	1,00		

Legend: N=number, p= significance level,OR=odds ratio, CI= confidence Interval, AN= anorexia nervosa, ICD-10=WHO´s International classification of Diseases, 10th edition, F50.1= atypical anorexia nervosa, EDE= Eating Disorder Examination, global EDE= global score of psychological symptoms derived from the EDE, BMI=Body mass index , relative BMI= actual BMI divided by the population-based median BMI for sex and age
