# Supplementary material for: Predictors of response to family-based treatment for anorexia nervosa in youth: insights from the VIBUS project
Source: Eur Child Adolesc Psychiatry. 2025 Jun 11;34(11):3665–84. doi: 10.1007/s00787-025-02766-x (PMC12647301; doi:10.1007/s00787-025-02766-x)
Supplement: Supplementary file 4 — Supplementary file4 (PDF 453 KB) [file 787_2025_2766_MOESM4_ESM.pdf]

Online Resource 4 for the manuscript: **Family Based Treatment for anorexia nervosa: Trajectories of improvement and characteristics of those who do not benefit sufficiently - A longitudinal study**

European Child & Adolescent Psychiatry

Mette Bentz, Signe Holm Pedersen, Ulla Moslet, Nikolaj Petersen, Anne Katrine Pagsberg
Correspondence: mette.bentz(at)regionh.dk, Child and Adolescent Mental Health Centre, Mental Health Services in the Capital Region of Denmark, Bispebjerg Bakke 30, DK 2400 Copenhagen NV

Supplementary Table: Significance and effect sizes of all the individually tested variables in analysis 2b (4 week predictors of time to successful treatment completion)(comma as decimal separator)

Covariates	N in analysis	level	N in level	n in level with succesful completion	p-value	estimate	lower CI limit	upper CI limit
Pharmacological Treatment	581	Yes	33	12	0,89	0,96	0,53	1,74
Pharmacological Treatment	581	No	548	313	0,89	1,00		
Illness insight*	560				0,01	0,83	0,72	0,96
Motivation for change*	560				<0.01	0,71	0,61	0,83
Collaborating relationship between YP and therapist*	559				0,10	0,88	0,76	1,02
Collaborating relationship between parents and therapist*	560				0,01	0,75	0,60	0,94
Number of menstrual cycles during last 3 months	521	None	370	201	<0.01	0,58	0,43	0,78
Number of menstrual cycles during last 3 months	521	>1	151	93	<0.01	1,00		
Restraint over eating (5-point likert scale)	542				<0.01	0,78	0,72	0,85
Desire to lose weight	542	1	155	66	<0.01	0,62	0,46	0,83
Desire to lose weight	542	0	387	243	<0.01	1,00		
Maintained low weight	517	1	308	160	0,02	0,75	0,59	0,95
Maintained low weight	517	0	209	133	0,02	1,00		
Binge eating episodes	581	Yes	29	14	0,37	1,30	0,75	2,23
Binge eating episodes	581	No	552	311	0,37	1,00		
Vomiting during last 4 weeks	581	Yes	33	22	0,54	0,87	0,55	1,37
Vomiting during last 4 weeks	581	No	548	303	0,54	1,00		

Misuse of laxantia during last 4 weeks	581	Yes	5	4	0,16	2,23	0,82	6,03
Misuse of laxantia during last 4 weeks	581	No	576	321	0,16	1,00		
Compulsive excercise during last 4 weeks	581	Yes	162	73	<0.01	0,58	0,44	0,75
Compulsive excercise during last 4 weeks	581	No	419	252	<0.01	1,00		
Importance of shape**	537				0,01	0,89	0,82	0,97
Importance of weight**	532				<0.01	0,87	0,81	0,93
Feeling fat during last 4 weeks, 7-point Likert-scale	540				<0.01	0,90	0,85	0,95
YP in school / work full time	581	Unchecked	387	203	0,23	0,86	0,67	1,10
YP in school / work full time	581	Checked	194	122	0,23	1,00		
YP in school / work part time	581	Unchecked	440	229	0,37	0,89	0,70	1,14
YP in school / work part time	581	Checked	141	96	0,37	1,00		
YP at leave from school / work	581	Unchecked	409	248	0,12	1,23	0,94	1,61
YP at leave from school / work	581	Checked	172	77	0,12	1,00		
YP meets with peers outside of school	581	Unchecked	263	133	0,08	0,82	0,65	1,03
YP meets with peers outside of school	581	Checked	318	192	0,08	1,00		
YP has structured activities outside of school	581	Unchecked	454	244	0,22	0,85	0,65	1,10
YP has structured activities outside of school	581	Checked	127	81	0,22	1,00		
One parent on leave to care for YP, part time	581	Unchecked	547	310	0,11	1,49	0,88	2,50
One parent on leave to care for YP, part time	581	Checked	34	15	0,11	1,00		
One parent on leave to care for YP, full time	581	Unchecked	367	205	0,65	1,05	0,83	1,33
One parent on leave to care for YP, full time	581	Checked	214	120	0,65	1,00		
Parents adviced to but not taking leave	581	Unchecked	504	275	0,13	0,78	0,58	1,07
Parents adviced to but not taking leave	581	Checked	77	50	0,13	1,00		
Quality of life during last 4 weeks, 5 point Likert-scale	523				0,00	0,81	0,72	0,91
Parents take on a leading role in renourishment*	581	Unchecked	193	90	0,87	0,98	0,76	1,26
Parents take on a leading role in renourishment*	581	Checked	388	235	0,87	1,00		
Parents take on a leading role in preventing eating disordered behaviours*	581	Unchecked	300	158	0,49	1,08	0,86	1,35
Parents take on a leading role in preventing eating disordered behaviours*	581	Checked	281	167	0,49	1,00		
Parents´ teamwork*	581	Unchecked	292	157	0,98	1,00	0,80	1,24
Parents´ teamwork*	581	Checked	289	168	0,98	1,00		
Parents able to help YP through difficult emotions*	581	Unchecked	309	161	0,47	1,09	0,87	1,36
Parents able to help YP through difficult emotions*	581	Checked	272	164	0,47	1,00		
YP is able to accept parental support*	581	Unchecked	244	118	0,12	0,83	0,66	1,05
YP is able to accept parental support*	581	Checked	337	207	0,12	1,00		
YP is able to take co-responsibility for working against AN	581	Unchecked	346	173	<0.01	0,61	0,48	0,76
YP is able to take co-responsibility for working against AN	581	Checked	235	152	<0.01	1,00		

Other factors enhancing treatment*	581	Unchecked	571	322	0,02	3,02	0,96	9,46
Other factors enhancing treatment*	581	Checked	10	3	0,02	1,00		
Difficult for parents to take a leading role in renourishment of YP*	581	Unchecked	465	279	0,01	1,47	1,07	2,02
Difficult for parents to take a leading role in renourishment of YP*	581	Checked	116	46	0,01	1,00		
Parents less able to help YP through difficult emotions	581	Unchecked	474	284	<0.01	1,74	1,24	2,44
Parents less able to help YP through difficult emotions	581	Checked	107	41	<0.01	1,00		
Parental collaboration is challenged*	581	Unchecked	531	305	0,10	1,44	0,91	2,27
Parental collaboration is challenged*	581	Checked	50	20	0,10	1,00		
difficult for YP to take co-responsibility for working against AN*	581	Unchecked	429	264	<0.01	1,64	1,23	2,19
difficult for YP to take co-responsibility for working against AN*	581	Checked	152	61	<0.01	1,00		
Other stressors on the family inhibit treatment*	581	Unchecked	524	307	0,08	1,51	0,93	2,46
Other stressors on the family inhibit treatment*	581	Checked	57	18	0,08	1,00		
YP comorbidity challenges treatment progress*	581	Unchecked	536	314	0,66	0,86	0,44	1,69
YP comorbidity challenges treatment progress*	581	Checked	45	11	0,66	1,00		
Other mental health issues in YP*	581	Unchecked	511	297	0,33	0,81	0,54	1,23
Other mental health issues in YP*	581	Checked	70	28	0,33	1,00		
YP challenged regarding emotion regulation/flexibility/Perfectionism*	581	Unchecked	472	274	0,43	1,13	0,83	1,53
YP challenged regarding emotion regulation/flexibility/Perfectionism*	581	Checked	109	51	0,43	1,00		
Challenges regarding YP school og peers*	581	Unchecked	499	292	0,45	1,15	0,79	1,67
Challenges regarding YP school og peers*	581	Checked	82	33	0,45	1,00		
Challenged relationship bwetween YP and parents*	581	Unchecked	550	318	0,21	1,57	0,73	3,36
Challenged relationship bwetween YP and parents*	581	Checked	31	7	0,21	1,00		
Conflicting views on treatment aproach, need of weight gain or other*	581	Unchecked	551	315	0,41	1,29	0,69	2,43
Conflicting views on treatment aproach, need of weight gain or other*	581	Checked	30	10	0,41	1,00		
Other treatment challenges*	581	Unchecked	536	297	0,02	0,62	0,42	0,92
Other treatment challenges*	581	Checked	45	28	0,02	1,00		

Legend: *= assessed by therapist, **=question borrowed from Eating Disorder Examination child version, CI=cinfidence interval, YP=young person, AN=anorexia nervosa
