# Supplementary material for: Predictors of response to family-based treatment for anorexia nervosa in youth: insights from the VIBUS project
Source: Eur Child Adolesc Psychiatry. 2025 Jun 11;34(11):3665–84. doi: 10.1007/s00787-025-02766-x (PMC12647301; doi:10.1007/s00787-025-02766-x)
Supplement: Supplementary file 5 — Supplementary file5 (PDF 460 KB) [file 787_2025_2766_MOESM5_ESM.pdf]

Online Resource 5 for the manuscript: **Family Based Treatment for anorexia nervosa: Trajectories of improvement and characteristics of those who do not benefit sufficiently - A longitudinal study**

European Child & Adolescent Psychiatry

Mette Bentz, Signe Holm Pedersen, Ulla Moslet, Nikolaj Petersen, Anne Katrine Pagsberg
Correspondence: mette.bentz(at)regionh.dk, Child and Adolescent Mental Health Centre, Mental Health Services in the Capital Region of Denmark, Bispebjerg Bakke 30, DK 2400 Copenhagen NV

Supplementary Table: Significance and effect sizes of all the individually tested variables in analysis 3 (Predictors for intensified treatment) (comma as decimal separator)

Covariates	n in analysis	level	p-value	n in level	OR	OR lower CI limit	OR upper CI limit	parameter estimate	Parameter estimate lower CI limit	Parameter estimate upper CI limit
Sex	652	Male	0,341	43	1	1	1	0	0	0
Sex	652	Female	0,341	610	1,48	0,68	3,69	0,39	-0,38	1,30
Age at start, continous	652		0,142		0,92	0,82	1,03	-0,08	-0,19	0,03
Age at start, discrete agegroups	652	<13	0,302	117	1	1	1	0	0	0
Age at start, discrete agegroups	652	13-15	0,302	272	0,70	0,43	1,17	-0,35	-0,85	0,16
Age at start, discrete agegroups	652	15-18	0,302	264	0,68	0,41	1,14	-0,38	-0,88	0,13
Compulsive exercise	617	No	0,609	213	1	1	1	0	0	0
Compulsive exercise	617	Yes	0,609	405	1,11	0,74	1,70	0,11	-0,30	0,53
Compulsive exercise, if yes, no. of days during last 4 weeks	355		0,403		0,99	0,96	1,01	-0,01	-0,04	0,01
Compulsive exercise, if yes, no. of days during last 4 weeks, discrete groups	594	>15	0,905	202	1	1	1	0	0	0
Compulsive exercise, if yes, no. of days during last 4 weeks, discrete groups	594	0	0,905	262	0,95	0,60	1,51	-0,05	-0,51	0,41
Compulsive exercise, if yes, no. of days during last 4 weeks, discrete groups	594	1-15	0,905	131	1,07	0,62	1,83	0,07	-0,48	0,60
Atypical AN (F50.1)	652	F50,0	<0,001	365	1	1	1	0	0	0
Atypical AN (F50.1)	652	F50,1	<0,001	288	0,39	0,26	0,59	-0,93	-1,35	-0,53
Atypical AN (ICD-10: F50.1) due to a smaller weight loss	652	Checked	<0,001	127	1	1	1	0	0	0
Atypical AN (ICD-10: F50.1) due to a smaller weight loss	652	Unchecked	<0,001	526	3,53	1,93	7,14	1,26	0,66	1,97
Atypical AN (ICD-10: F50.1) due to avoidance of fattening foods	652	Checked	0,046	8	1	1	1	0	0	0
Atypical AN (ICD-10: F50.1) due to avoidance of fattening foods	652	Unchecked	0,046	645	1640173,76	0,00		14,31	-29,28	
Atypical AN (ICD-10: F50.1) due to not feeling fat	652	Checked	0,195	74	1	1	1	0	0	0
Atypical AN (ICD-10: F50.1) due to not feeling fat	652	Unchecked	0,195	579	1,51	0,82	3,02	0,41	-0,20	1,11
Atypical AN (ICD-10: F50.1) due to no endocrine disturbances (i.e.; girls still menstruating)	652	Checked	0,027	117	1	1	1	0	0	0
Atypical AN (ICD-10: F50.1) due to no endocrine disturbances (i.e.; girls still menstruating)	652	Unchecked	0,027	536	1,81	1,07	3,23	0,59	0,06	1,17
Atypical AN (ICD-10: F50.1) due to presence of bulimic symptoms	652	Checked	0,428	41	1	1	1	0	0	0
Atypical AN (ICD-10: F50.1) due to presence of bulimic symptoms	652	Unchecked	0,428	612	1,39	0,64	3,48	0,33	-0,45	1,25
History of bullying in general	601	Yes	0,300	115	1	1	1	0	0	0
History of bullying in general	601	No	0,300	487	1,32	0,79	2,32	0,28	-0,24	0,84

Other adversities related to peer relationships prior to AN debut	652	Checked	0,926	167	1	1	1	0	0	0
Other adversities related to peer relationships prior to AN debut	652	Unchecked	0,926	486	0,98	0,65	1,51	-0,02	-0,44	0,41
Relational challenges in the family prior to AN debut	652	Checked	0,647	85	1	1	1	0	0	0
Relational challenges in the family prior to AN debut	652	Unchecked	0,647	568	1,14	0,66	2,07	0,13	-0,41	0,73
Practical, economic, or health-related adversities in the family prior to AN debut	652	Checked	0,340	39	1	1	1	0	0	0
Practical, economic, or health-related adversities in the family prior to AN debut	652	Unchecked	0,340	614	0,70	0,35	1,50	-0,36	-1,06	0,40
Larger changes in e.g., housing or school prior to AN debut	652	Checked	0,070	133	1	1	1	0	0	0
Larger changes in e.g., housing or school prior to AN debut	652	Unchecked	0,070	520	0,66	0,43	1,03	-0,41	-0,84	0,03
A history of bullying prior to AN debut	652	Checked	0,897	24	1	1	1	0	0	0
A history of bullying prior to AN debut	652	Unchecked	0,897	629	1,07	0,42	3,27	0,07	-0,87	1,18
A history of overweight prior to AN debut	652	Checked	0,815	34	1	1	1	0	0	0
A history of overweight prior to AN debut	652	Unchecked	0,815	619	0,91	0,42	2,18	-0,10	-0,87	0,78
Mental health issues in child prior to AN debut	652	Checked	0,094	78	1	1	1	0	0	0
Mental health issues in child prior to AN debut	652	Unchecked	0,094	575	0,63	0,38	1,08	-0,46	-0,98	0,08
Somatic health issues in child prior to AN debut	652	Checked	0,659	15	1	1	1	0	0	0
Somatic health issues in child prior to AN debut	652	Unchecked	0,659	638	0,77	0,26	2,80	-0,27	-1,36	1,03
Loss or deaths of close others prior to AN debut	652	Checked	0,041	22	1	1	1	0	0	0
Loss or deaths of close others prior to AN debut	652	Unchecked	0,041	631	0,39	0,16	0,96	-0,94	-1,81	-0,04
Other adversities prior to AN debut	652	Checked	0,155	205	1	1	1	0	0	0
Other adversities prior to AN debut	652	Unchecked	0,155	448	1,35	0,90	2,06	0,30	-0,11	0,72
Prior interventions from municipal authorities	204	Yes	0,014	48	1	1	1	0	0	0
Prior interventions from municipal authorities	204	No	0,014	156	0,40	0,19	0,83	-0,92	-1,64	-0,19
Traumas affecting the family	205	Yes	0,114	12	1	1	1	0	0	0
Traumas affecting the family	205	No	0,114	193	0,37	0,11	1,29	-1,01	-2,20	0,26
Somatic or mental health issues in siblings	208	Yes	0,721	24	1	1	1	0	0	0
Somatic or mental health issues in siblings	208	No	0,721	184	0,83	0,32	2,42	-0,18	-1,12	0,88
Somatic or mental illness in a parent to a degree deemed affecting their ability to take on an active role in renourishment	206	Yes	0,509	14	1	1	1	0	0	0
Somatic or mental illness in a parent to a degree deemed affecting their ability to take on an active role in renourishment	206	No	0,509	192	0,66	0,21	2,50	-0,42	-1,57	0,92
Clinician´s assessment of mother's ability to take an active role in renourishment, 3-point Likert scale	596		0,001		1,65	1,23	2,20	0,50	0,21	0,79
Clinician´s assessment of father's ability to take an active role in renourishment 3-point Likert scale	577		0,536		1,10	0,81	1,46	0,09	-0,21	0,38
Binging and/or purging behaviours	642	Binging and purging	0,951	26	1	1	1	0	0	0
Binging and/or purging behaviours	642	No	0,951	487	0,89	0,37	2,50	-0,11	-0,99	0,91
Binging and/or purging behaviours	642	Only binging	0,951	52	1,00	0,33	3,23	0,00	-1,09	1,17
Binging and/or purging behaviours	642	Only purging	0,951	78	0,79	0,28	2,47	-0,23	-1,27	0,90
EDE global score	632		0,005		1,23	1,06	1,43	0,21	0,06	0,35
Duration of restrictive eating before start, reported by young person	482		0,430		0,99	0,96	1,01	-0,01	-0,04	0,01

Parents´ own assessment or their ability to take an active role in renourishment	575	Yes	0,022	503	1	1	1	0	0	0
Parents´ own assessment or their ability to take an active role in renourishment	575	No	0,022	4	4,35	0,52	36,64	1,47	-0,66	3,60
Parents´ own assessment or their ability to take an active role in renourishment	575	In doublt	0,022	69	2,04	1,15	3,51	0,71	0,14	1,26
Cargiver status	637	Living apart, both active	0,549	127	1	1	1	0	0	0
Cargiver status	637	One responsible parent	0,549	47	1,57	0,73	3,32	0,45	-0,32	1,20
Cargiver status	637	Other caregiver	0,549	3	1,85	0,08	20,04	0,62	-2,47	3,00
Cargiver status	637	parents living together	0,549	461	0,97	0,61	1,60	-0,03	-0,50	0,47
Mental retardation or intellectualis inferioritas	652	No	0,314	645	1	1	1	0	0	0
Mental retardation or intellectualis inferioritas	652	Yes	0,314	8	2,16	0,44	8,93	0,77	-0,82	2,19
Autism spectrum (ICD-10: F80-88)	652	No	0,000	517	1	1	1	0	0	0
Autism spectrum (ICD-10: F80-88)	652	Yes	0,000	136	3,31	2,19	5,00	1,20	0,78	1,61
Behavioral emotional disorder (ICD-10: F90-98)	652	No	0,014	604	1	1	1	0	0	0
Behavioral emotional disorder (ICD-10: F90-98)	652	Yes	0,014	49	2,23	1,18	4,07	0,80	0,17	1,40
Affective disorders (ICD-10: F30-38)	652	No	0,001	619	1	1	1	0	0	0
Affective disorders (ICD-10: F30-38)	652	Yes	0,001	34	3,44	1,69	6,95	1,24	0,53	1,94
Anxiety disorders (ICD-10: F40-48)	652	No	0,015	600	1	1	1	0	0	0
Anxiety disorders (ICD-10: F40-48)	652	Yes	0,015	53	2,15	1,16	3,85	0,76	0,15	1,35
Other comorbidities	652	No	0,121	641	1	1	1	0	0	0
Other comorbidities	652	Yes	0,121	12	2,60	0,76	8,28	0,96	-0,27	2,11
relative BMI at intake	652		<0,001		0,00	0,00	0,03	-5,52	-7,62	-3,52
relative BMI, start of treatment, discrete groups	652	<0.8	<0,001	226	1	1	1	0	0	0
relative BMI, start of treatment, discrete groups	652	>1	<0,001	51	0,10	0,02	0,33	-2,31	-4,14	-1,10
relative BMI, start of treatment, discrete groups	652	0.8-1	<0,001	376	0,60	0,41	0,89	-0,50	-0,89	-0,12

Legend:N=number, p= significance level,OR=odds ratio, CI= confidence Interval, AN= anorexia nervosa, ICD-10=WHO´s International classification of Diseases, 10th edition, F50.1= atypical anorexia nervosa, EDE= Eating Disorder Examination, global EDE= global score of psychological symptoms derived from the EDE, BMI=Body mass index , relative BMI= actual BMI divided by the population-based median BMI for sex and age, *=assesment by therapist
